# Supplementary material for: Incidence of acute lower respiratory tract disease hospitalisations, including pneumonia, among adults in Bristol, UK, 2019, estimated using both a prospective and retrospective methodology
Source: BMJ Open. 2022 Jun 15;12(6):e057464. doi: 10.1136/bmjopen-2021-057464 (PMC9204403; doi:10.1136/bmjopen-2021-057464)
Supplement: Supplementary data [file bmjopen-2021-057464supp001.pdf]

Supplementary Data 1: ICD-10 codes used for patient identification for Retrospective Analysis

ICD-10 code	Definition	Pneumonia	NP-LRTI	HF	Other aLRTD
J09	Influenza due to identified zoonotic or pandemic influenza virus		X		
J10	Influenza due to identified seasonal influenza virus		X		
J11	Influenza, virus not identified		X		
J12	Viral pneumonia, not elsewhere classified	X			
J13	Pneumonia due to *Streptococcus pneumoniae*	X			
J14	Pneumonia due to *Haemophilus influenzae*	X			
J15	Bacterial pneumonia, not elsewhere classified	X			
J16	Pneumonia due to other infectious organisms, not elsewhere classified	X			
J17	Pneumonia in diseases classified elsewhere	X			
J18	Pneumonia, organism unspecified	X			
J20	Acute bronchitis		X		
J21	Acute bronchiolitis		X		
J22	Unspecified acute lower respiratory infection		X		
J40	Bronchitis, not specified as acute or chronic		X		
J41	Simple and mucopurulent chronic bronchitis		X		
J42	Unspecified chronic bronchitis		X		
J43	Emphysema				X
J44	Other chronic obstructive pulmonary disease (including J44.0 chronic		J44.0 only		X (J44.0)

	obstructive pulmonary disease with acute lower respiratory infection)				
J45	Asthma				X
J46	Status asthmaticus				X
J47	Bronchiectasis				X
J85	Abscess of lung and mediastinum		X		
J86	Pyothorax		X		
J90	Pleural effusion, not elsewhere classified				X
J91	Pleural effusion in conditions classified elsewhere				X
J95	Postprocedural respiratory disorders, not elsewhere classified				X
J96	Respiratory failure, not elsewhere classified				X
J98	Other respiratory disorders				X
J99	Respiratory disorders in diseases classified elsewhere				X
I110	Hypertensive heart disease with heart failure			X	
I130	Hypertensive heart and chronic kidney disease with heart failure and stage 1 through stage 4 chronic kidney disease			X	
I132	Hypertensive heart and chronic kidney disease with heart failure and with stage 5 chronic kidney disease, or end stage renal disease			X	
I50	Heart failure			X	
