# Supplementary material for: Incidence of acute lower respiratory tract disease hospitalisations, including pneumonia, among adults in Bristol, UK, 2019, estimated using both a prospective and retrospective methodology
Source: BMJ Open. 2022 Jun 15;12(6):e057464. doi: 10.1136/bmjopen-2021-057464 (PMC9204403; doi:10.1136/bmjopen-2021-057464)
Supplement: Supplementary data [file bmjopen-2021-057464supp002.pdf]

Supplementary Data 2: Case Definitions

**Condition**	**Definition**	**Reference**
Acute Lower Respiratory Tract Disease (aLRTD)	Acute lower respiratory tract disease (aLRTD) encompasses pneumonia, non-pneumonic lower respiratory tract infection (LRTI), acute bronchitis, exacerbation of underlying respiratory disease including asthma and chronic obstructive pulmonary disease (COPD), and cardiac failure with respiratory symptoms.<br><br>Pneumothorax, pulmonary embolism, progression or new diagnosis of primary or secondary lung malignancy were excluded from aLRTD.	
Pneumonia	Pneumonia was defined as infection affecting the airways (below the level of the larynx), with either:<br>(1) an acute illness with radiographic shadowing which was at least segmental or present in more than one lobe and was not known to be previously present or due to other causes<br>(2) in the absence of radiological investigation, clinical confirmation of pneumonic disease in the opinion of the treating physician	Lim WS, Baudouin SV, George RC, *et al*; Pneumonia Guidelines Committee of the BTS Standards of Care Committee. BTS guidelines for the management of community acquired pneumonia in adults: update 2009. Thorax. 2009 Oct;64 Suppl 3:iii1-55.
Non-Radiologically Proven Lower Respiratory Tract Infection (NP-LRTI)	An infection that affects the airways (below the level of the larynx) including the trachea and alveoli, with neither the presence of radiological change nor a clinical diagnosis of pneumonia from the treating physician, i.e. non-pneumonic infection in the lungs.	Anderson W, Winter J. Managing LRTI in adults in the community. Practitioner. 2009 Nov;253(1723):21-5, 2-3. PMID: 20043506.
Cardiac/Heart Failure (HF)	A clinical syndrome with symptoms and/or signs caused by a structural and/or functional cardiac abnormality and corroborated by elevated natriuretic peptide levels and or objective evidence of pulmonary or systemic congestion.	Bozkurt, Biykem et al.Universal Definition and Classification of Heart Failure. Journal of Cardiac Failure, Volume 27, Issue 4, 387 – 413.
Other aLRTD	aLRTD which was neither pneumonia, NP-LRTI nor HF was classified as 'Other aLRTD'. This therefore includes non-infective exacerbations of chronic respiratory disease such as asthma, COPD and bronchiectasis	
