# Supplementary material for: Incidence of acute lower respiratory tract disease hospitalisations, including pneumonia, among adults in Bristol, UK, 2019, estimated using both a prospective and retrospective methodology
Source: BMJ Open. 2022 Jun 15;12(6):e057464. doi: 10.1136/bmjopen-2021-057464 (PMC9204403; doi:10.1136/bmjopen-2021-057464)
Supplement: Author's manuscript [file bmjopen-2021-057464.draft_revisions.pdf]

BMJ Open is committed to open peer review. As part of this commitment we make the peer review history of every article we publish publicly available.

When an article is published we post the peer reviewers' comments and the authors' responses online. We also post the versions of the paper that were used during peer review. These are the versions that the peer review comments apply to.

The versions of the paper that follow are the versions that were submitted during the peer review process. They are not the versions of record or the final published versions. They should not be cited or distributed as the published version of this manuscript.

BMJ Open is an open access journal and the full, final, typeset and author-corrected version of record of the manuscript is available on our site with no access controls, subscription charges or pay-per-view fees (http://bmjopen.bmj.com).

If you have any questions on BMJ Open's open peer review process please email info.bmjopen@bmj.com

BMJ Open

Incidence of Hospitalized Acute Lower Respiratory Tract Disease Including Pneumonia and Lower Respiratory Tract Infection in Bristol, England, 2019

Journal:	*BMJ Open*
Manuscript ID	bmjopen-2021-057464
Article Type:	Original research
Date Submitted by the Author:	18-Sep-2021

Complete List of Authors:	Hyams, Catherine; University of Bristol, Academic Respiratory Unit; University of Bristol, School of Population Health Sciences<br>Begier, Beth; Pfizer Inc<br>Garcia Gonzalez, Maria; University of Bristol; Southmead Hospital, Academic Respiratory Unit<br>Southern, Jo; Pfizer Ltd<br>Campling, James; Pfizer Ltd<br>Gray, Sharon; Pfizer Inc<br>Oliver, Jennifer; University of Bristol, Schools of Cellular and Molecular Medicine and Population Health Sciences<br>Gessner, Bradford; Pfizer Inc<br>Finn, Adam; University of Bristol, Schools of Cellular and Molecular Medicine and Population Health Sciences

Keywords:	Heart failure < CARDIOLOGY, Respiratory infections < THORACIC MEDICINE, Epidemiology < INFECTIOUS DISEASES

SCHOLARONE™
Manuscripts

I, the Submitting Author has the right to grant and does grant on behalf of all authors of the Work (as defined in the below author licence), an exclusive licence and/or a non-exclusive licence for contributions from authors who are: i) UK Crown employees; ii) where BMJ has agreed a CC-BY licence shall apply, and/or iii) in accordance with the terms applicable for US Federal Government officers or employees acting as part of their official duties; on a worldwide, perpetual, irrevocable, royalty-free basis to BMJ Publishing Group Ltd ("BMJ") its licensees and where the relevant Journal is co-owned by BMJ to the co-owners of the Journal, to publish the Work in this journal and any other BMJ products and to exploit all rights, as set out in our licence.

The Submitting Author accepts and understands that any supply made under these terms is made by BMJ to the Submitting Author unless you are acting as an employee on behalf of your employer or a postgraduate student of an affiliated institution which is paying any applicable article publishing charge ("APC") for Open Access articles. Where the Submitting Author wishes to make the Work available on an Open Access basis (and intends to pay the relevant APC), the terms of reuse of such Open Access shall be governed by a Creative Commons licence – details of these licences and which Creative Commons licence will apply to this Work are set out in our licence referred to above.

Other than as permitted in any relevant BMJ Author's Self Archiving Policies, I confirm this Work has not been accepted for publication elsewhere, is not being considered for publication elsewhere and does not duplicate material already published. I confirm all authors consent to publication of this Work and authorise the granting of this licence.

For peer review only

Title: Incidence of Hospitalized Acute Lower Respiratory Tract Disease Including Pneumonia and Lower Respiratory Tract Infection in Bristol, England, 2019

Authors: Catherine Hyams[1,2,3], Elizabeth Begier[4], Maria Garcia Gonzalez[1,2], Jo Southern[5], James Campling[5], Sharon Gray[4], Jennifer Oliver[1], Bradford D. Gessner[4], Adam Finn[1]

Affiliations:

[1] Bristol Vaccine Centre, Schools of Cellular and Molecular Medicine and Population Health Sciences, University of Bristol, Bristol, BS2 8AE, United Kingdom

[2] Academic Respiratory Unit, Learning and Research Building, Southmead Hospital, Bristol, UK, BS10 5NB

[3] Acute Medical Unit, Southmead Hospital, Bristol, UK, BS10 5NB

[4] Vaccines Medical Development, Scientific and Clinical Affairs, Pfizer Inc, Collegeville, PA, USA

[5] Vaccines Medical Affairs, Pfizer Ltd, Tadworth, UK, KT20 7NS

Corresponding author: Catherine Hyams, MBBS BSc(Hons) PhD
Population Health Sciences, University of Bristol,
Bristol, BS2 8AE,
United Kingdom
catherine.hyams@nbt.nhs.uk
Catherine.hyams@bristol.ac.uk

Alternate author: Maria Garcia Gonzalez
qa18884@bristol.ac.uk

Running Title: Incidence of acute lower respiratory tract disease in adults

Keywords: Pneumonia, respiratory infection, lower respiratory tract infection, heart failure

Funding: CH was funded by the National Institute for Health Research (NIHR) [NIHR Academic Clinical Fellowship (ACF-2015-25-002]. The views expressed are those of the author(s) and not necessarily those of the NIHR or the Department of Health and Social Care. The remainder of the study funding was from Pfizer, Inc (WI255886-1).

For peer review only - http://bmjopen.bmj.com/site/about/guidelines.xhtml

Competing Interests: EB, JS, JC, SG, and BG are full time employees of Pfizer Vaccines and hold stock or stock options. CH is Principal Investigator of the Avon CAP study which is an investigator-led University of Bristol study funded by Pfizer and has previously received support from the NIHR in an Academic Clinical Fellowship. AF is a member of the Joint Committee on Vaccination and Immunization (JCVI) and chair of the World Health Organization European Technical Advisory Group of Experts on Immunization (ETAGE) committee. In addition to receiving funding from Pfizer as Chief Investigator of this study, he leads another project investigating transmission of respiratory bacteria in families jointly funded by Pfizer and the Gates Foundation. The other authors have no relevant conflicts of interest to declare.

Word count: **2997/4000**

Summary (38/40 words): Using both retrospective and prospective methodologies, we found a high annual incidence of acute lower respiratory disease (>1700 per 100000; 1.7%) in and around Bristol, UK: pneumonia (~0.6%), lower respiratory tract infection without pneumonia (>0.7%), and heart failure (>0.3%).

Author Contributions: CH, EB, MGG, JS, BDG and AF generated the research questions and analysis plan CH, EB, MGG, JS, JC, SG and BDG undertook and contributed to the data analysis. AF oversaw the research and data collection which was undertaken by CH and MGG. All authors contributed to the preparation of the manuscript.

For peer review only

Abstract (278/300):

Objectives

To determine the disease burden of aLRTD and its subsets (pneumonia, lower respiratory tract infection [LRTI], heart failure) in hospitalized adults in Bristol, UK.

Setting

Single-centre, secondary care hospital, Bristol UK

Design

We estimated aLRTD hospitalizations incidence in adults (≥18 years) in Bristol, UK using two approaches. First, retrospective ICD-10 code analysis (first five positions/hospitalization) identified aLRTD events over a 12-month period (March2018–Feb2019). Secondly, during a 21-day prospective review (19Aug–9Sept2019), aLRTD admissions were identified, categorized by diagnosis, and subsequently annualized. Hospital catchment denominators were calculated using linked general practice and hospitalization data, with each practice's denominator contribution calculated based on practice population and percent of the practices' hospitalizations admitted to the study hospital.

Participants

Prospective review: 1322 adults screened; 410 identified with aLRTD. Retrospective review: 7727 adult admissions.

Primary and Secondary outcome measures

The incidence of aLRTD and its subsets in the adult population of Southmead Hospital, Bristol UK.

Results

Based on ICD-10 code analysis, annual incidences per 100000 population were: aLRTD, 1901; pneumonia, 591; LRTI, 739; heart failure, 402. aLRTD incidence was highest among those ≥65-years: 65–74 (3684 per 100000 adults), 75–84 (6962 per 100000 adults), and ≥85 (11430 per 100000 adults).

For peer review only - http://bmjopen.bmj.com/site/about/guidelines.xhtml

During the prospective review, 410/1322 (31%) hospitalized adults had aLRTD signs/symptoms, and annualized incidences closely replicated retrospective analysis results.

Conclusions

aLRTD disease burden was high, increasing sharply with age. aLRTD incidence is probably higher than estimated previously due to criteria specifying respiratory-specific symptoms or radiological change, usage of only the first diagnosis code, and mismatch between case count sources and population denominators. This may have significant consequences for healthcare planning, including usage of current and future vaccinations against respiratory infection.

Strengths and Limitations of This Study

- We used two analytical methods at the same site over a comparable period, to calculate incidence using both prospective and retrospective approaches.
- The case burden of aLRTD and its subgroups was pre-defined and included patients with atypical presentations
- We calculated incidence using a denominator derived from GP records, providing increased accuracy compared to population calculations based on census data.
- This was a single-center study, with a predominantly Caucasian cohort; therefore, the findings might not be generalizable to other populations.
- The ICD-10 coding data analysis was limited to codes within the first five positions, and therefore may have excluded some cases where other diagnoses were placed higher in the diagnostic coding hierarchy.

For peer review only - http://bmjopen.bmj.com/site/about/guidelines.xhtml

Introduction

Acute lower respiratory tract disease (aLRTD) encompasses pneumonia, lower respiratory tract infection (LRTI), acute bronchitis, exacerbation of underlying respiratory diseases, including asthma and chronic obstructive pulmonary disease (COPD), and acute heart failure (HF) events resulting in respiratory symptoms (e.g. breathlessness). Before the COVID-19 pandemic, European healthcare costs for pneumonia alone were estimated at €10 billion annually, including €5.7 billion for inpatient care. [1] Pneumonia incidence in Europe varies by country and intra-country region, age, socioeconomic status, and gender; [2-4, 7] however, in all studies pneumonia incidence increases sharply with age. [3] Pneumonia affects an estimated 0.5% to 1% of UK adults each year [5, 6]. Overall LRTI incidence is considerably higher with ~15% of UK adults aged ≥65 years experiencing an event each year. [7]

However, aLRTD incidence may be considerably higher than previously reported, given that published literature has documented several reasons why previous estimates may have been erroneously low. Estimates of aLRTD based on pneumonia defined as radiologically demonstrated alveolar infiltrates, may underestimate true disease burden as chest radiography (CXR) is an imperfect gold-standard. Immunosuppressed, elderly or dehydrated patients are likely to be under-represented if respiratory infection is defined by radiologically demonstrated changes. [8, 9] Microbiological investigations for pneumonia are undertaken variably and identify a causative pathogen in 50% of cases at most [10, 11]; hence, the disease is probably under-reported when confirmed microbiological diagnosis is required. Furthermore, Respiratory Syncytial Virus (RSV) infection has recently been recognized as an important respiratory pathogen later in life, [16] with severe disease occurring in patient groups in whom the diagnosis is likely to be under-recognized (e.g. the elderly or those with underlying cardiac conditions). [17] Studies of clinical coding data are retrospective and subject to recognized limitations associated with this methodology. [12, 13] Older patients with pneumonia often have atypical

For peer review only - http://bmjopen.bmj.com/site/about/guidelines.xhtml

presenting signs and symptoms, which may lead to missed or incorrect admission diagnoses. [14] Pneumonia may occur secondary to, or be an underlying cause of, the main presenting complaint, particularly in patients with cerebrovascular accidents (CVA), heart failure (HF), COPD exacerbations or altered consciousness levels. [15] In these scenarios, pneumonia may not be the primary hospitalization diagnosis code and may not even be coded as an associated diagnosis.

Given the paucity of data supporting accurate aLRTD incidence rates and its disease subsets, we undertook to assess aLRTD incidence by two approaches (retrospective and prospective) in Bristol, UK, seeking to determine the disease burden of hospitalized aLRTD and its subgroups more accurately.

For peer review only

For peer review only - http://bmjopen.bmj.com/site/about/guidelines.xhtml

Methods

Study Design

This study was conducted at a large secondary care institution in UK (North Bristol NHS Trust) with specialist Respiratory Services (interstitial lung disease, pleural disease). Two approaches were undertaken to estimate aLRTD incidence: (1) "retrospective analysis" of aLRTD International Classification of Diseases Tenth Revision (ICD-10) diagnostic codes for an entire year; and, (2) 21-day observational "prospective review" of aLRTD hospital admissions.

Ethics

This study was approved by North Bristol NHS Trust Research Audit Ethics Committee (CA52218).

Patient and Public Involvement

No patient involved.

Retrospective Analysis

For the retrospective analysis, all adult inpatient admissions (≥18 years) to the study hospital during March 2018–February 2019 with aLRTD ICD-10 diagnostic codes (Supplementary data 1) in any of the first 5 positions were identified and categorized into aLRTD subgroups: pneumonia, LRTI, other LRTD, and HF. A mutually exclusive hierarchy was used (pneumonia, LRTI, then other LRTD) although HF diagnoses could co-occur with other categories. "Other LRTD" included acute respiratory events that could not definitively be placed in another category.

Prospective Review

Adult patients (≥18 years) resident within Bristol, North Somerset, and South Gloucestershire Clinical Commissioning Group (CCG) referred to the Acute Medical Unit (AMU) at North Bristol NHS Trust during 19th August –9th September 2019 were included. A respiratory physician (CH) reviewed presenting features and investigation results for each admitted patient to determine whether aLRTD

For peer review only - http://bmjopen.bmj.com/site/about/guidelines.xhtml

106 was present. Further medical record review was undertaken if patients had: new/worsening
107 breathlessness, cough or sputum production; new or worsening peripheral oedema; pleurisy; clinical
108 examination findings consistent with respiratory infection or HF; or fever attributable to suspected
109 respiratory infection. Patients with non-respiratory diagnoses were excluded.

110 *Prospective Review Outcome measures*

111 aLRTD was considered confirmed in individuals with: new/worsening respiratory symptoms, with or
112 without fever; sepsis, delirium or raised inflammatory markers attributable to likely respiratory
113 infection in admitting clinical team's opinion; radiological change in keeping with infection (e.g.
114 consolidation); and/or final diagnosis of LRTI, pneumonia or infective exacerbation of a chronic
115 respiratory condition. A pneumonia diagnosis was assigned if radiological changes likely due to
116 infection were described by the reporting radiologist. A LRTI diagnosis was assigned if aLRTD signs and
117 symptoms likely to be due to infection were present without demonstrated radiological change. A HF
118 diagnosis was assigned in presence of: new/worsening breathlessness and bi-basal crepitations,
119 cardiac wheeze or new/worsening bilateral pitting oedema; elevated pro-NT BNP (≥450pg/mL);
120 radiologist-reported radiographic changes consistent with cardiac failure; or a final consultant
121 physician diagnosis of HF, cardiac failure or left-ventricular failure. When present, ≥1 diagnosis was
122 selected.

123 For both retrospective and prospective studies, pneumonia included both community and healthcare
124 setting acquired cases; although, the prospective review only captured admitting diagnoses and
125 pneumonias occurring later during hospitalization were not included.

126 *Incidence calculations*

127 Annual incidence per 100000 persons was calculated for both retrospective and prospective studies.
128 Case counts from prospective review were annualized (i.e., case counts by diagnosis and overall were

For peer review only

For peer review only - http://bmjopen.bmj.com/site/about/guidelines.xhtml

divided by the percentage of annual admissions for these diagnosis groups that occurred in this 21-day period in the retrospective analysis).

Incidence Denominators

To calculate appropriate population denominators for incidence calculations, aLRTD hospital admission event data were linked to aggregated GP practice patient registration data within the NHS Bristol, North Somerset, and South Gloucestershire CCG for 2017–2019. Only 4% of patients sought care at North Bristol NHS Trust hospital from outside these local CCGs, despite presence of specialist respiratory services. For GP practices within these same CCGs, the proportion of their aLRTD admissions occurring at North Bristol NHS Trust was multiplied by their patient registration count in 2019 by age group, to get each practice's contribution to the denominator (e.g. if 50% aLRTD admissions were at North Bristol among persons 50−64 years, the practice would contribute half of their patients 50–64 years to the denominator). Further details of this methodology have been described previously. [27]

Statistical analysis

Patient characteristics were tabulated by aLRTD diagnosis. Categorical variables were presented as counts with percentages. Continuous data are presented with means and standard deviations (SD) if normally distributed and medians and interquartile range (IQR) if not normally distributed. Patient groups difference were evaluated using the Friedman test with Wilcoxon signed-rank test.

For peer review only - http://bmjopen.bmj.com/site/about/guidelines.xhtml

Results

Retrospective analysis

Over a 12-month period, we identified 7727 hospital admissions for aLRTD: 3005 LRTI admissions, 2402 pneumonia, 1633 HF and 1071 other LRTD (Table 1). aLRTD admissions were lowest in March and April and highest December through February (Figure 1A), overall and for all aLRTD subgroups (P<0.05) (Figure 1B-D). Overall, 28.1% (2244) cases were identified as being potentially hospital-acquired infection based on co-occurring ICD-10 code 'Y95 Nosocomial Infection'.

Prospective review

Among 1322 eligible adult patients referred to AMU over the 21-day review period (Figure 2), 410 patients had signs or symptoms of aLRTD: 188 (46%) LRTI; 152 (37%) pneumonia, and 77 (19%) HF. Seven patients had both decompensated HF and a respiratory infection at hospital admission. On admission, >10% of aLRTD patients did not have respiratory symptoms: 16 (11%) pneumonia, 25 (13%) LRTI, and 18 (14%) HF (Table 2).

Almost all adults admitted with aLRTD underwent routine biochemistry, hematology, and radiological investigation (99.9%, n=409). In contrast, only 150 (37%) patients with aLRTD had microbiological testing performed: blood cultures (n=149, 36%) and urine cultures (n=143, 35%). Pneumonia patients more commonly underwent microbiological investigation than LRTI patients (P<0.05) with highest disparity in rates of sputum culture, urinary antigens, and respiratory viral PCR (Table 2). All cardiac failure patients who underwent microbiological investigation had concomitant respiratory infection (Table 2). Overall, a microbiological diagnosis was found in 11 (3%) cases, highlighting the low frequency of definitive pathogen identification in aLRTD. Younger patients underwent microbiological testing more frequently than the elderly for all aLRTD categories (Table 2).

For peer review only - http://bmjopen.bmj.com/site/about/guidelines.xhtml

Disease incidence

Retrospective analysis yielded an overall aLRTD incidence of 1901 per 100000. Disease incidence rose with increasing age (Table 3), both overall and for all disease subgroups; incidences per 100000 among adults aged ≥85 years were: 11430 (aLRTD), 6116 (LRTI), 4215 (pneumonia) and 4005 (HF). Overall, 28.1% aLRTD hospitalizations also included an ICD-10 discharge code for 'nosocomial infection', suggesting the aLRTD event or other infection was hospital-acquired. For pneumonia 25.3% had the nosocomial infection code. If all of these were related to the pneumonia diagnosis, an estimated residual 1794 events would have been community-acquired pneumonia (annual incidence 441/100000 adults). Among older age categories, where the incidence of aLRTD was highest, LRTI incidence was similar to pneumonia incidence, with an approximate 1:1 ratio of LRTI to pneumonia cases. However, among adults under age 50 years, there were approximately twice as many LRTI cases observed as pneumonia cases. Incidence calculations using annualized prospective review results were broadly comparable with retrospective analysis of ICD-10 data (Table 3).

For peer review only - http://bmjopen.bmj.com/site/about/guidelines.xhtml

Discussion:

This is the first UK study to assess aLRTD incidence comprehensively. We conducted an analysis of 12-months of hospital admissions by ICD-10 diagnosis code data and a 21-day prospective review at a large academic hospital in southwest England. With both approaches, we found a high annual incidence of aLRTD (>1700 per 100000; 1.7%), pneumonia (~0.6%), LRTI without pneumonia (>0.7%), and HF (>0.4%). Incidences increased sharply in a non-linear manner as age increased above 65 years for all aLRTD categories. These results suggest rates are probably significantly higher than previous disease estimates from the UK (Table 4) but comparable with many results globally, [18,19] with important consequences for healthcare resources. For example, a recent review highlighted that pneumonia incidences ranged from 1000 to 2500 per 100000 (1–2.5%) among persons aged 65–74 years in Spain, Germany, France, Japan and the US, which are comparable to the >1250 per 100000 (1.3%) reported here. Some of the potential sources of underestimation for other UK incidence studies (Table 4) include: case source and denominator mismatch; use of first position in ICD-code analysis only; inclusion of enrolled subjects in consent-based prospective studies only; requiring specific symptoms and chest x-ray confirmation in order to be classified as pneumonia/aLRTD; and, lastly, the rising incidence of aLRTD.

Comparison with published literature

No studies have reported aLRTD incidence comprehensively in UK hospitalized patients within the last 20 years. However, eight publications report incidence of ≥1 aLRTD subgroup. Seven publications reported community-acquired pneumonia (CAP) incidence (three from Nottingham, UK). For pneumonia, our incidence estimates were three to four-fold higher than other UK inpatient incidence estimates (Table 4) but comparable to estimates from other countries. [18, 19] Only two UK studies from approximately 20 years ago reported LRTI incidence (one with both CAP and LRTIs; Table 4), and only one provided an inpatient estimate. [25] LRTI incidence was approximately 2-fold lower than that

For peer review only - http://bmjopen.bmj.com/site/about/guidelines.xhtml

calculated here, taking into account inclusion of CAP and other LRTI in their estimates. [25] The one UK study reporting HF incidence had methodological differences (i.e., inclusion of outpatients and limiting to initial HF diagnosis) and estimates could not be compared. [26] Close examination of the existing literature methods yielded multiple sources for potential underestimation.

First, for incidence studies that were not countrywide, identifying an appropriate denominator is challenging. Like many other inpatient settings worldwide, UK hospitals' catchment areas for acute treatment are principally driven by geography, but the proportion of any area's residents expected to use the hospital becomes less clear as distance from the hospital increases because catchment areas and populations of different hospitals may overlap. Defining hospital catchment populations based solely on census data cannot account for this variability. Including all geographic areas using the hospital to any extent results in population denominator overestimation and underestimated incidence. Here, we addressed this by calculating population denominators based on hospital utilization behavior from referring General Practices.

Second, other studies used fewer codes in their ICD code analysis: all limited their analyses to events where the diagnostic code was in the first position (Table 4; case definition column), potentially excluding admissions in which pneumonia/LRTI complicated other underlying respiratory diseases, including COPD and asthma. Limiting to first position has been shown to reduce sensitivity for pneumonia events by about 30% (66%–72% sensitive). [20, 21] Conversely, the recent British Thoracic Society (BTS) audit on CAP found ~27% of pneumonia events identified by ICD code (J12-18) had no new CXR infiltrates. [6] Even accounting for this potential over coding practice, our estimates remain well above other published UK estimates.

Third, for other prospective studies, exclusion of events where patients did not consent to participation or were not identified by study surveillance processes (often conducted predominately during business hours) can introduce underestimation. Further, other prospective pneumonia studies specifically required documentation of specific symptoms, radiological findings, and treatments, [22]

For peer review only - http://bmjopen.bmj.com/site/about/guidelines.xhtml

potentially excluding those without these features documented in medical records. In our prospective review, approximately 11% did not display typical signs and symptoms of pneumonia and could have been excluded by that requirement. Requiring CXR confirmation has been shown to reduce incidence estimates for pneumonia, [19] although all pneumonia events in our prospective review were radiologically confirmed.

Fourth, trends over time may also contribute to our estimates being higher than previous reports. Our study's estimates are recent, and rising incidence of pneumonia has been documented in all studies that have reported such trends. [22-24]

Finally, this study included, in part, hospital-acquired pneumonias (HAP), which were excluded from estimates calculated in some other studies (Table 4). The retrospective analysis may have included more nosocomial infection than the prospective review, as the latter was focused on evaluation of patients at admission for aLRTD and would not have reliably captured events that developed during hospitalization. 25.3% pneumonia events included a nosocomial infection code, but this code could relate to any nosocomial infection during that hospitalization. If all these cases were assumed to be hospital-acquired pneumonia, our estimates community-acquired pneumonia incidence would still be well above prior UK estimates: 441/100000 (≥18 years).

While not impacting all-cause aLRTD incidence estimates discussed above, during the prospective review, we found low rates of microbial investigation which prevented us from generating pathogen-specific incidence estimates. Only 52% of patients with radiologically-confirmed pneumonia underwent microbiological testing during hospitalization, with even lower rates in other aLRTD subgroups (41% LRTI and 14% HF). Microbiological testing occurred less frequently as age increased, particularly in LRTI patients. It is possible that, because aLRTD hospitalizations are substantially more common among older persons, less etiologic investigation is performed. Furthermore, clinicians may elect to treat elderly patients with a more pragmatic and less invasive approach. Management guidelines do not require specific pathogen identification to inform treatment choice. Presence only

For peer review only - http://bmjopen.bmj.com/site/about/guidelines.xhtml

of atypical features on presentation (in this series, 13% LRTI and 11% pneumonia cases) may also reduce the likelihood of timely microbiological testing. Low rates of microbiological testing, and consequently of confirmed microbiological diagnosis, may represent a source of underestimation of pathogen-specific disease incidence in patient groups (i.e., testing bias), particularly in elderly patient groups.

Strengths and Limitations of This Study

This study has many strengths. First, this study used two analytical methods at the same site over a comparable period, to calculate incidence using both prospective and retrospective approaches. Second, the case burden of aLRTD and its subgroups was pre-defined and included patients with atypical presentations but with clinical and/or radiological diagnoses, who may otherwise have been excluded from analysis. Additionally, we calculated incidence using a denominator derived from GP records, providing increased accuracy compared to population calculations based on census data.

However, the study also had some limitations. This was a single-center study, with a predominantly Caucasian cohort; therefore, the findings might not be generalizable to other populations. The ICD-10 coding data analysis was limited to codes within the first five positions, and therefore may have excluded some cases where other diagnoses were placed higher in the diagnostic coding hierarchy. Furthermore, we could not determine how many cases of the 28.1% ICD-10 cases also coded with nosocomial infection had hospital-acquired respiratory infection rather than other nosocomial infections.

Although the denominator used to calculate incidence was derived from GP records, this was still an estimate as there is no precisely defined denominator hospital catchment. We were unable to exclude patients from outside the local CCGs in the retrospective analysis, due to the way ICD-10 data was obtained. However, these patients were excluded from the prospective review and the incidence

For peer review only - http://bmjopen.bmj.com/site/about/guidelines.xhtml

calculated was comparable, suggesting any effect that patients attending North Bristol NHS Trust from outside the local CCGs have on incidence estimates is minimal. This may be because any effect of travelling or health-seeking behavior is bi-directional: whilst some patients admitted to Southmead hospital were from outside the local area, it is also true that patients with aLRTD within the relevant CCGs would have been admitted to other hospitals. We also acknowledge the 21-day prospective review period was relatively short, not repeated and may not be fully representative of clinical practice and cases throughout the year.

In conclusion, we found similarly high estimates of LRTD incidence using two different approaches. Combining all types of LRTD highlights the high burden for this important and potentially life-threatening disease group. Incidence assessments require close assessments of potential areas of under ascertainment, including unidentified or unenrolled cases in prospective studies, reduced positions or number of ICD-10 codes included for retrospective studies, and population denominator mismatch for all study types. Our prospective review findings highlight the need to consider atypical clinical presentations for pneumonia and the lack of routine microbiological investigation in many aLRTD patients required for pathogen-specific aLRTD incidence calculation. Future research should include a fully prospective assessment of aLRTD incidence with comprehensive diagnostic testing across multiple respiratory seasons, to ensure accurate capture of all aLRTD events, particularly in the elderly. Such research should be undertaken given the high and rising aLRTD burden to enable appropriate healthcare planning and identification of interventions which may reduce disease burden.

For peer review only - http://bmjopen.bmj.com/site/about/guidelines.xhtml

Acknowledgements

We would like to acknowledge the assistance of Qi Yan, PhD (Pfizer, Inc.) who provided indispensable medical writing and literature review support for this manuscript and Harvey Walsh Health who performed the hospital denominator calculation used here. For the denominator analysis, Hospital Episode Statistics (HES) Data were re-used with the permission of NHS Digital via Harvey Walsh Limited.

Data Sharing

No additional data available

For peer review only

References

1. Gibson GJ, Loddenkemper R, Lundback B, Sibille Y. Respiratory health and disease in Europe: the new European Lung White Book. Eur Respir J 2013; 42(3): 559-63.
2. Cilloniz C, Polverino E, Ewig S, et al. Impact of age and comorbidity on cause and outcome in community-acquired pneumonia. Chest **2013**; 144(3): 999-1007.
3. Torres A, Cilloniz C, Blasi F, et al. Burden of pneumococcal community-acquired pneumonia in adults across Europe: A literature review. Respir Med **2018**; 137: 6-13.
4. European Lung White Book. Pneumonia. Available at: https://www.erswhitebook.org/chapters/acute-lower-respiratory-infections/pneumonia/#:~:text=pneumonia%20(VAP).-,Incidence,000%20cases%20are%20expected%20annually. Accessed April 25, 2021.
5. British Lung Foundation. Pneumonia Statistics. Available at: https://statistics.blf.org.uk/pneumonia. Accessed April 25, 2021.
6. Lim W, Lawrence H. British Thoracic Society. National Audit Report: Adult Community Acquired Pneumonia Audit 2018-2019. National Audit Period: 1 December 2018 – 31 January 2019, **2019**.
7. Millett ER, Quint JK, Smeeth L, et al. Incidence of community-acquired lower respiratory tract infections and pneumonia among older adults in the United Kingdom: a population-based study. PLoS One **2013**; 8(9): e75131.
8. Maughan BC, Asselin N, Carey JL, et al. False-negative chest radiographs in emergency department diagnosis of pneumonia. R I Med J (2013) **2014**; 97(8): 20-3.
9. Self WH, Courtney DM, McNaughton CD, et al. High discordance of chest x-ray and computed tomography for detection of pulmonary opacities in ED patients: implications for diagnosing pneumonia. Am J Emerg Med **2013**; 31(2): 401-5.
10. Cilloniz C, Ewig S, Polverino E, et al. Microbial aetiology of community-acquired pneumonia and its relation to severity. Thorax **2011**; 66(4): 340-6.
11. Jain S, Self WH, Wunderink RG, Team CES. Community-Acquired Pneumonia Requiring Hospitalization. N Engl J Med **2015**; 373(24): 2382.
12. Mainor AJ, Morden NE, Smith J, et al. ICD-10 Coding Will Challenge Researchers: Caution and Collaboration may Reduce Measurement Error and Improve Comparability Over Time. Med Care **2019**; 57(7): e42-e6.
13. Burns EM, Rigby E, Mamidanna R, et al. Systematic review of discharge coding accuracy. J Public Health (Oxf) **2012**; 34(1): 138-48.

For peer review only

For peer review only - http://bmjopen.bmj.com/site/about/guidelines.xhtml

14. Henig O, Kaye KS. Bacterial Pneumonia in Older Adults. Infect Dis Clin North Am **2017**; 31(4): 689-713.

15. Fine MJ, Smith MA, Carson CA, et al. Prognosis and outcomes of patients with community-acquired pneumonia. A meta-analysis. JAMA **1996**; 275(2): 134-41.

16. Falsey AR, Walsh EE. Respiratory syncytial virus infection in elderly adults. Drugs Aging **2005**; 22(7): 577-87.

17. Branche AR, Falsey AR. Respiratory syncytial virus infection in older adults: an under-recognized problem. Drugs Aging **2015**; 32(4): 261-9.

18. Chalmers J, Campling J, Ellsbury G, Hawkey PM, Madhava H, Slack M. Community-acquired pneumonia in the United Kingdom: a call to action. Pneumonia (Nathan) **2017**; 9: 15.

19. Shi T, Denouel A, Tietjen AK, et al. Global and Regional Burden of Hospital Admissions for Pneumonia in Older Adults: A Systematic Review and Meta-Analysis. J Infect Dis **2020**; 222(Suppl 7): S570-S6.

20. van de Garde EM, Oosterheert JJ, Bonten M, et al. International classification of diseases codes showed modest sensitivity for detecting community-acquired pneumonia. J Clin Epidemiol **2007**; 60(8): 834-8.

21. Yu O, Nelson JC, Bounds L, Jackson LA. Classification algorithms to improve the accuracy of identifying patients hospitalized with community-acquired pneumonia using administrative data. Epidemiology and infection **2011**; 139(9): 1296-306.

22. Pick H, Daniel P, Rodrigo C, et al. Pneumococcal serotype trends, surveillance and risk factors in UK adult pneumonia, 2013-18. Thorax **2020**; 75(1): 38-49.

23. Elston JW, Santaniello-Newton A, Meigh JA, et al. Increasing incidence of invasive pneumococcal disease and pneumonia despite improved vaccination uptake: surveillance in Hull and East Yorkshire, UK, 2002-2009. Epidemiology and infection **2012**; 140(7): 1252-66.

24. Thorrington D, Andrews N, Stowe J, Miller E, van Hoek AJ. Elucidating the impact of the pneumococcal conjugate vaccine programme on pneumonia, sepsis and otitis media hospital admissions in England using a composite control. BMC medicine **2018**; 16(1): 13.

25. Lovering AM, MacGowan AP, Anderson P, Irwin D. Epidemiology and resource utilization for patients hospitalized for lower respiratory tract infection. Clin Microbiol Infect **2001**; 7(12): 666-70.

26. Uijl A, Koudstaal S, Direk K, et al. Risk factors for incident heart failure in age- and sex-specific strata: a population-based cohort using linked electronic health records. European journal of heart failure **2019**; 21(10): 1197-206.

For peer review only

For peer review only - http://bmjopen.bmj.com/site/about/guidelines.xhtml

27. Campling J, Begier E, Vyse A, et al. A novel approach to calculate the local population denominator to calculate disease incidence for hospital admission related health events in England. Abstract for: Public Health Science: A National Conference Dedicated to New Research in UK Public Health. 26 November, 2021.

For peer review only

For peer review only - http://bmjopen.bmj.com/site/about/guidelines.xhtml

Table 1: Demographic Characteristics of Patients admitted with aLRTD for 1 Year ICD-10 code Retrospective Analysis and 21-day Prospective Review Period -

- 2018–2019

Characteristic	Pneumonia		LRTI		Heart Failure		Other LRTD	All LRTD	
Study	Prospective review	Retrospective analysis	Prospective review	Retrospective analysis	Prospective review	Retrospective analysis	Retrospective Review Only	Prospective review	Retrospective analysis
N	152	2402	188	3005	77	1633	1071	410	7727
Gender, females	61 (40)	1078 (45)	99 (53)	1482 (49)	39 (51)	731 (45)	489 (46)	194 (47)	3780 (49)
**Age**									
Median (IQR), y	80 (67-86)	81 (66-88)	70 (46-87)	69 (45-87)	87 (72-90)	87 (70-90)	74 (53-82)	80 (64-88)	81 (65-90)
18-24	4 (3)	72 (3)	9 (5)	151 (5)	0 (0)	0 (0)	26 (2)	13 (3)	249 (3)
25-34	2 (2)	48 (2)	12 (6)	183 (6)	0 (0)	3 (0)	33 (3)	14 (3)	267 (3)
35-44	6 (4)	97 (4)	14 (7)	209 (7)	2 (3)	10 (1)	59 (6)	22 (5)	375 (5)
45-54	8 (5)	118 (5)	11 (6)	183 (6)	0 (0)	22 (1)	112 (10)	19 (5)	435 (6)
55-64	18 (12)	293 (12)	19 (10)	305 (10)	8 (10)	158 (10)	210 (20)	45 (11)	966 (13)
65-74	34 (22)	501 (21)	32 (17)	549 (18)	10 (15)	199 (12)	223 (21)	75 (18)	1472 (19)
75-84	44 (28)	667 (28)	40 (21)	621 (21)	20 (30)	498 (31)	205 (19)	100 (24)	1991 (26)
≥85	38 (26)	606 (25)	51 (27)	704 (23)	37 (55)	742 (45)	203 (19)	123 (30)	2255 (29)

For peer review only - http://bmjopen.bmj.com/site/about/guidelines.xhtml

Table 2. Clinical Characteristics and Investigations of Patients admitted with aLRTD over 21-day Prospective Review Period in August-September 2020

**Characteristic**	**Pneumonia n=152 (%)**	LRTI n=188 (%)	Heart Failure n=77 (%)	All LRTD n=410 (%)
GP	56 (37)	72 (39)	30 (39)	158 (39)
A&E department	93 (61)	100 (54)	45 (58)	238 (58)
Transfer from another unit	2 (1)	13 (7)	0 (0)	15 (4)
Other	1 (1)	1 (1)	2 (3)	4 (1)
**Referral Source**				
Typical features†	136 (89)	163 (87)	63 (82)	355 (87)
Atypical features	16 (11)	25 (13)	14 (18)	55 (13)
- collapse/falls	11 (7)	12 (6)	0 (0)	23 (6)
- confusion	0 (0)	7 (4)	4 (5)	10 (2)
- drowsiness	1 (1)	1 (1)	2 (3)	4 (1)
- off legs/generally unwell	5 (3)	5 (3)	8 (10)	18 (4)
**LRTD Signs and symptoms on referral to AMU**				
Biochemistry	152 (100)	185 (99)	77 (100)	419 (100)
Haematology	152 (100)	185 (99)	77 (100)	419 (100)
Radiology	152 (100)	185 (99)	77 (100)	419 (100)
**Investigations Performed**				
Testing by Age Group				
All patients	79/152 (52)*	77/188 (41)	11/77 (14)	167 (41)
18-24	3/4 (75)	6/9 (66)	0/0 (0)	9/13 (69)
25-34	0/0 (0)	6/12 (50)	0/0 (0)	6/12 (50)
35-44	5/6 (83)	10/14 (71)	2/2 (100)	17/22 (77)
45-54	6/8 (75)	6/11 (55)	0/0 (0)	13/19 (68)
55-64	11/18 (61)	12/19 (63)	5/8 (63)	31/45 (69)
65-74	15/34 (44)	12/32 (38)	1/10 (10)	28/75 (37)
75-84	21/43 (49)	10/40 (25)	2/20 (10)	33/100 (33)
≥85	18/39 (46)	15/51 (19)	1/37 (3)	34/124 (27)
Test performed				
Blood culture	79 (52)	70 (37)	5 (6)	150 (37)
Urine culture	66 (43)	77 (41)	11 (14)	150 (37)
Sputum culture	27 (18)*	7 (4)	2 (3)	35 (9)
BinaxNOW® Pn UAT ‡	29 (19)*	6 (3)	0 (0)	35 (9)
Respiratory virus PCR	16 (11)*	11 (6)	1 (1)	28 (7)
Pleural fluid culture	3 (2)	0 (0)	1 (1)	4 (1)

A&E, Accident and Emergency department; GP, general practitioner; LRTD, lower respiratory tract disease; LRTI, lower respiratory tract infection; IQR, interquartile range; Pn UAT, pneumococcal urinary antigen test; PCR, polymerase chain reaction.
* *P*<0.05.
† Typical symptoms included cough, breathlessness, increased or discolored sputum production, wheeze, pleurisy, peripheral oedema, hemoptysis, reduced exercise tolerance and/or fever.
‡ BinaxNOW Pn UAT was only performed in accordance with NICE/BTS guidelines.

For peer review only

For peer review only - http://bmjopen.bmj.com/site/about/guidelines.xhtml

Table 3: Incidence of aLRTD resulting in hospital admission based on prospective and retrospective approaches by age group and condition, North Bristol National Health Service Trust— United Kingdom 2018–2019.

	Age Groups					
	**All Adults**	**18-49 y**	**50-64 y**	**65-74 y**	**75-84 y**	**≥85 y**
**Population in 2018**	406481	226920	91534	45705	29487	12835
**Retrospective Analysis of a Year's ICD-10 codes**						
**Annual cases – N (row %)**						
All aLRTD	7,727	1,130 (14)	1,103 (14)	1,684 (22)	2,053 (27)	1,757 (23)
Pneumonia	2,402	264 (11)	288 (12)	589 (25)	720 (30)	541 (22)
LRTI	3,005	576 (19)	410 (14)	572 (19)	662 (22)	785 (26)
Other LRTD	1,071	246 (23)	268 (25)	226 (21)	200 (19)	131 (12)
Heart Failure	1,633	48 (3)	189 (12)	397 (24)	485 (30)	514 (31)
**LRTI/Pneumonia Ratio**	1.3	2.2	1.4	1.0	0.9	1.5
**Incidence (per 100,000)**						
All aLRTD	1,901	497	1,205	3,684	6,962	13,689
Pneumonia	591	116	315	1,289	2,442	4,215
LRTI	739	254	448	1,252	2,245	6,116
Other LRTD	263	108	293	494	678	1,021
Heart Failure	402	21	206	869	1,645	4,005
**21-day Prospective Review (annualized)**						
**Annualized cases – N (row %)**						
All aLRTD	7,885	1,038	962	1,692	2,231	1,962
Pneumonia	2,621	224	397	776	690	534
LRTI	3,857	796	531	653	1,061	816
Heart Failure	2,000	51	205	308	641	795
**LRTI/Pneumonia Ratio**	1.5	3.6	1.3	0.8	1.5	1.5
**Incidence (per 100,000)**						
All aLRTD	1,940	458	1,050	3,703	7,565	15,283
Pneumonia	645	99	433	1,698	2,339	4,164
LRTI	944	351	580	1,429	3,599	6,360
Heart Failure	492	23	224	673	2,174	6,193

LRTI = lower respiratory tract infection; LRTD = lower respiratory tract disease.
Pneumonia category includes community and healthcare acquired. For retrospective ICD-10 based cohort, the following mutually exclusive hierarchy was used to define pneumonia, LRTI, and other LRTD; heart failure event could overlap with other categories. "Other LRTD" contains LRTD events that could not definitively be placed in one of the other respiratory disease categories.

For peer review only - http://bmjopen.bmj.com/site/about/guidelines.xhtml

Figure Legends:

Figure 1: aLRTD admissions identified by Retrospective ICD-10 diagnostic code analysis at North Bristol National Health Service Trust— United Kingdom 2018–2019.

Monthly number of patients admitted, based on ICD-10 coding analysis, with (A) all acute Lower Respiratory Tract Disease (aLRTD) (black bars), (B) Pneumonia (slashed bars), (C) Lower Respiratory Tract Infection (LRTI) (white bars), (D) Other LRTD (cross-hash bars), and (E) Heart Failure (grey bars).

Figure 2: Flow diagram of the Prospective Review

For peer review only

For peer review only - http://bmjopen.bmj.com/site/about/guidelines.xhtml

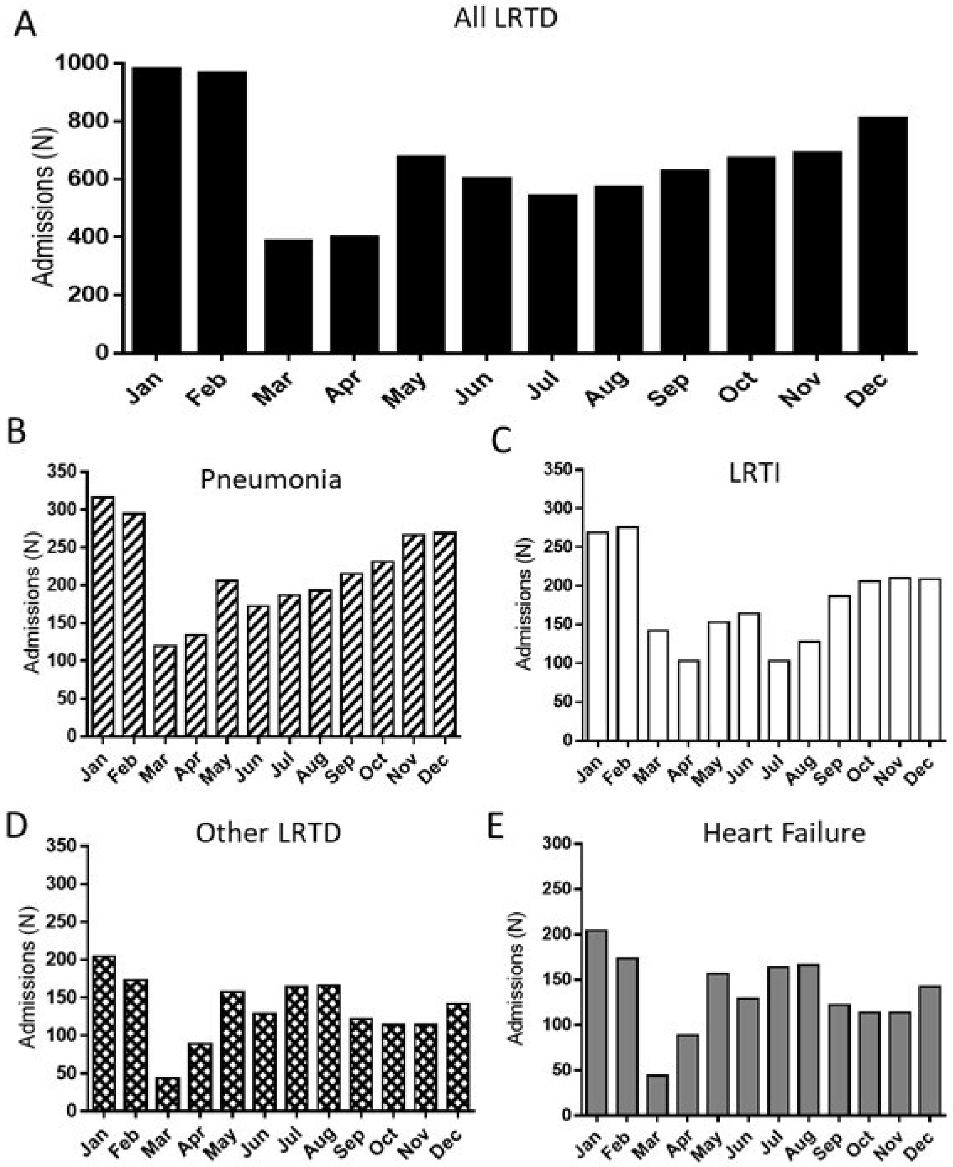

Figure 1: aLRTD admissions identified by Retrospective ICD-10 diagnostic code analysis at North Bristol National Health Service Trust— United Kingdom 2018–2019.
Monthly number of patients admitted, based on ICD-10 coding analysis, with (A) all acute Lower Respiratory Tract Disease (aLRTD) (black bars), (B) Pneumonia (slashed bars), (C) Lower Respiratory Tract Infection (LRTI) (white bars), (D) Other LRTD (cross-hash bars), and (E) Heart Failure (grey bars).

49x60mm (300 x 300 DPI)

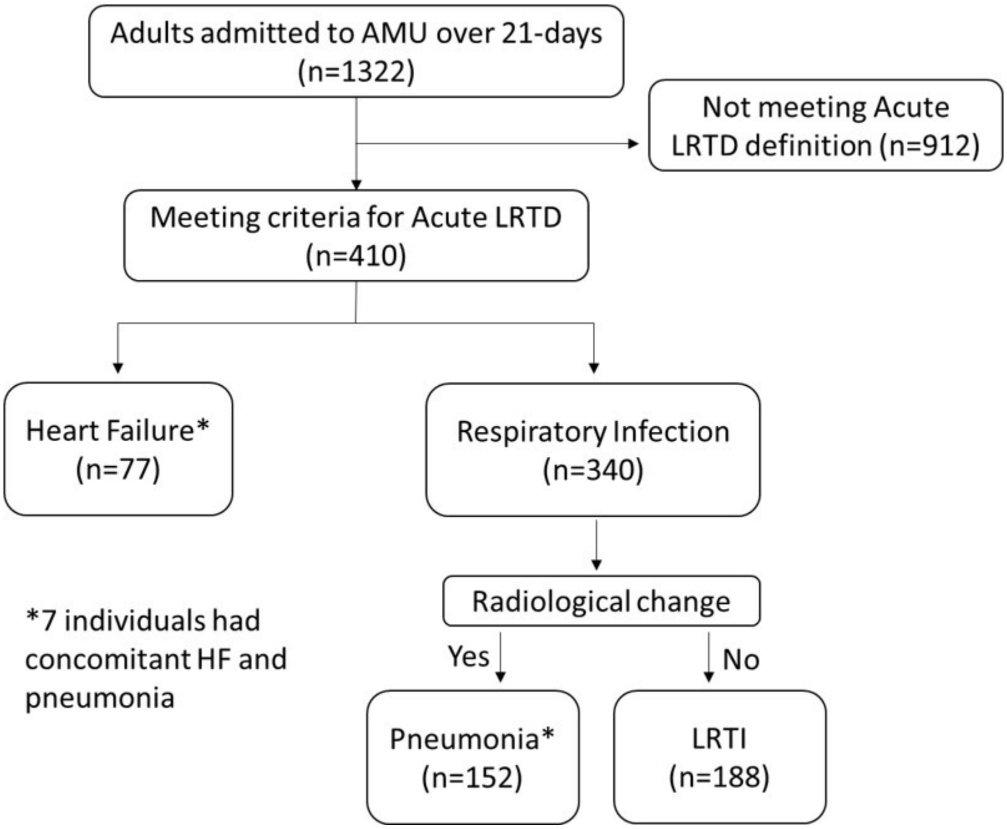

Figure 2: Flow diagram of the Prospective Review

67x55mm (300 x 300 DPI)

Table 4: Literature review of aLRTD incidence in hospitalized adults, United Kingdom

Study	Study Years	Location (Facility)	Event Setting	Age	Case Definition[b]	Key inclusion	Denominator Source	Overall Incidence	Age Breakdown (years)	Incidence per 100,000 by age[d]	Comments
**Community –acquired Pneumonia**											
Current Study	2018 – 2019	Bristol (Southmead Hospital)	Inpatients only	≥18 y	Clinical signs/symptoms with radiological change in keeping with infection (prospective review portion) **AND** Retrospective ICD-10 code analysis (1st 5 positions): J12-J18, J85, and J86	Hospital-acquired pneumonia (HAP) included	Based on number of persons ≥18 years registered in referring GP practices. For practices with split referral patterns, number adjusted for percent of admissions that came to Southmead.	648<br>591	18 –49<br>50 –64<br>65 –74<br>75 –84<br>≥85	116<br>315<br>1,289<br>2,442<br>4,215	Retrospective analysis includes 1st 5 positions
Elston 2012, Epidemiol Infect	2002 – 2009	Hull and East Yorkshire Hospitals[c]	Inpatients only	≥16 y	ICD-10 codes (1st position only): J18.0, J18.9, J13X, J18.1, and J15X	HAP included	Mid-year population estimates for Hull (city) and EroY (Surrounding County) from Office for National Statistics	143 (2002) – 207 (2009)	15 –64<br>≥65	48.8 – 84.1<br>543 – 781	Fewer ICD-10 codes included than other analyses; Y95 Nosocomial infection included.
Millet 2013, J Clin Epidemiol	1997 – 2011	UK	Both inpatients and outpatients	≥65 y	Read and ICD 10 codes; no specified codes provided. For ICD-10, used first diagnosis code for first episode of hospitalization only.	HAP Excluded	Mid-year UK population estimates from Office for National Statistics	799	65 –69<br>70 –74<br>75 –79<br>80 –84 y<br>85 –89<br>≥90	281<br>431<br>694<br>1,205<br>2,184<br>4,194	Incidence estimates converted to per 100,000 person-years
Pick 2020, Thorax[a]	2013 – 2014	Nottingham (2 large university hospitals)	Inpatients only	≥16 y	Inclusion criteria: one or more symptom suggestive of LRTI (defined as cough, increasing dyspnea, sputum production and fever), with evidence of acute infiltrates consistent with respiratory infection on admission radiography, and treated for a diagnosis of CAP Exclusion criteria: hospitalization within 10 days of index admission, a diagnosis of tuberculosis or post-obstructive pneumonia.	HAP Excluded	Mid-year estimates for the Greater Nottingham area from the Office for National Statistics, including local population data stratified by age group	96.3	16 –49<br>50 –64<br>65 –74<br>75 –84<br>≥85	27.3<br>80.2<br>181.3<br>400.6<br>707.5	Only consented/enrolled subjects included in estimates Required CXR -confirmation but not all LRTI patients had CXR Census-derived denominator that may not have fully matched catchment area.
	2017 – 2018							158.4	16 –49<br>50 –64	29.9<br>146.9	

For peer review only

	2013 – 2018							120.4	65 –74<br>75 –84<br>≥85<br>--	310.4<br>559.5<br>1522.6<br>--	Required specific symptoms and evidence of treatment and some CAP events may not have had this information documented
Thorrington 2019, BMC Med	2004 – 2005<br>2014 – 2015	England	Inpatients only	≥65 y	ICD 10 codes (1st position only): J18 (pneumonia of unspecified causative organism)	HAP Included	Mid-year population estimates for England for 2004 to 2015 from Office for National Statistics	NA	≥65<br>≥65	829<br>1787	Incidence is per 100,000 person-years. Fewer ICD-10 codes included than other analyses
Trotter 2008, EID	1997 – 1998<br>2004 – 2005	England	Inpatients only	≥65 y	ICD 10 codes (1st position only): J12 – J18	HAP Included	Mid-year population estimates for England for 1997 to 2004 from the Office for National Statistics	NA	65 –74<br>75 –84<br>≥85<br>65 –74<br>75 –84<br>≥85	263<br>684<br>1599<br>355<br>877<br>2218	Incidence estimates converted to 100,000 population
**Lower Respiratory Tract Infection**						**Pneumonia**					
Current Study	2018 – 2019	Bristol (Southmead Hospital)	Inpatients only	≥18 y	Clinical signs/symptoms of heart failure or elevated pro-NT BNP or radiological change<br>**AND**<br>Retrospective ICD-10 code analysis: J09, J10, J11; J20, J21, J22, J40, J41, J42, J44, J45, and J46	Excludes all Pneumonia	Based on number of persons ≥18 years registered in referring GP practices. For practices with split referral patterns, number adjusted for percent of admissions that came to Southmead.	802<br>739	18 –49<br>50 –64<br>65 –74<br>75 –84<br>≥85	254<br>448<br>1,252<br>2,442<br>6,116	
Lovering 2001, Clinical Micro. & Infection	1994 – 1996	Bristol (Southmead Hospital)	Inpatients only	≥16 y	LRTI episodes from four groups by ICD 9/10 codes: (1) CAP; (2) chest infection or acute exacerbation in presence of asthma; (3) chest infection or acute exacerbation in presence of COPD; or (4) bronchitis with no radiological evidence of pneumonia or pre-existing respiratory disease, such as COPD or asthma. No specified codes provided.	Includes Community-acquired Pneumonia<br>HAP Excluded	No information on denominator provided	623	16 –39<br>40 –49<br>50 –59<br>60 –69<br>70 –79<br>>79	151<br>175<br>294<br>1,086<br>2,135<br>3,141	Incidence converted to per 100,000 population<br>Study involved single hospital and no mention of source of denominator mentioned.
Millet	1997 –	UK	Both	≥65 y	Read and ICD 10 codes; no specified	Includes	Mid-year UK population	12,293	65 –69	9,221	Incidence

For peer review only

2013, J Clin Epidemiol	2011		inpatients and outpatients		codes provided. For ICD-10, used first diagnosis code for inpatient episode only.	Community-acquired Pneumonia<br><br>HAP Excluded	estimates from Office for National Statistics. (Patients were not considered at risk for community-acquired LRTI during an LRTI illness-episode, during a HES hospitalization, or for 14 days after any HES hospitalization or CPRD hospital code. This person-time was excluded from denominator.)		70 –74<br>75 –79<br>80 –84<br>85 –89<br>≥90	10,740<br>12,607<br>15,137<br>18,791<br>26,287	converted to per 100,000 person years
**Heart Failure**						**All or First Episode**					
Current Study	2018-2019	Bristol (Southmead Hospital)	Inpatients only	≥18 y	Clinical signs/symptoms of heart failure or elevated pro-NT BNP or radiological change<br><br>**AND**<br><br>Retrospective ICD-10 code analysis: I110; I130; I132; I50	All	Based on number of persons ≥18 years registered in referring GP practices. For practices with split referral patterns, number adjusted for percent of admissions that came to Southmead.	328<br><br>402	18 –49<br>50 –64<br>65 –74<br>75 –84<br>≥85	21<br>206<br>869<br>1,645<br>4,005	
Uijl 2019, Eur J Heart Fail	2000 – 2010	UK	Both inpatients and outpatients	≥55 y	4 sources of HER were linked: CPRD primary care records, HES secondary care hospital charges, Myocardial Ischaemia National Audit Project (MINAP) disease registry, and ONS national death registry. HES ICD-10 codes: Heart failure: I110, I130, I132, I260, I50 and I21. Individuals were excluded if they presented a history of HF before their index date in CPRD, HES or MINAP.	First episode at 55 years or older counted	Not Reported	Not Reported	55 –64, M<br>55 –64, F<br>65 –74, M<br>65 –74, F<br>>75, M<br>>75, F	360<br>190<br>1,360<br>920<br>3,440<br>2,800	Incidence converted to per 100,000 person years<br>Included first episode of HF (inpatient or outpatient) at age 55 and up, so repeat episodes not included.

[a] Only the most recent incidence estimates from the Nottingham CAP study were included.

[b] Add text names for all listed ICD codes in this footnote or appendix.

[c] Included hospitals were Hull Royal Infirmary, Castle Hill Hospital, Princess Royal Hospital, Scarborough District General Hospital, Bridlington & District Hospital, York Teaching Hospital, Scunthorpe General Hospital, Goole & District Hospital.

[d] For the current study, only age-group estimates from the retrospective analysis were included in this table, but these were closely comparable to the annualized incidence estimates based on the prospective review. See Table 3 for full results.

For peer review only

STROBE Statement—checklist of items that should be included in reports of observational studies

	Item No	Recommendation	Page No
**Title and abstract**	1	(*a*) Indicate the study's design with a commonly used term in the title or the abstract	3
		(*b*) Provide in the abstract an informative and balanced summary of what was done and what was found	3
**Introduction**			
Background/rationale	2	Explain the scientific background and rationale for the investigation being reported	5-6
Objectives	3	State specific objectives, including any prespecified hypotheses	6
**Methods**			
Study design	4	Present key elements of study design early in the paper	7
Setting	5	Describe the setting, locations, and relevant dates, including periods of recruitment, exposure, follow-up, and data collection	7-9
Participants	6	(*a*) *Cohort study*—Give the eligibility criteria, and the sources and methods of selection of participants. Describe methods of follow-up<br>*Case-control study*—Give the eligibility criteria, and the sources and methods of case ascertainment and control selection. Give the rationale for the choice of cases and controls<br>*Cross-sectional study*—Give the eligibility criteria, and the sources and methods of selection of participants	7-9
		(*b*) *Cohort study*—For matched studies, give matching criteria and number of exposed and unexposed<br>*Case-control study*—For matched studies, give matching criteria and the number of controls per case	
Variables	7	Clearly define all outcomes, exposures, predictors, potential confounders, and effect modifiers. Give diagnostic criteria, if applicable	8
Data sources/ measurement	8*	For each variable of interest, give sources of data and details of methods of assessment (measurement). Describe comparability of assessment methods if there is more than one group	7-8
Bias	9	Describe any efforts to address potential sources of bias	15-16
Study size	10	Explain how the study size was arrived at	7-8
Quantitative variables	11	Explain how quantitative variables were handled in the analyses. If applicable, describe which groupings were chosen and why	8-9
Statistical methods	12	(*a*) Describe all statistical methods, including those used to control for confounding	9
		(*b*) Describe any methods used to examine subgroups and interactions	NA
		(*c*) Explain how missing data were addressed	NA
		(*d*) *Cohort study*—If applicable, explain how loss to follow-up was addressed<br>*Case-control study*—If applicable, explain how matching of cases and controls was addressed<br>*Cross-sectional study*—If applicable, describe analytical methods taking account of sampling strategy	NA
		(*e*) Describe any sensitivity analyses	NA

Continued on next page

For peer review only - http://bmjopen.bmj.com/site/about/guidelines.xhtml

**Results**			
Participants	13*	(a) Report numbers of individuals at each stage of study—eg numbers potentially eligible, examined for eligibility, confirmed eligible, included in the study, completing follow-up, and analysed	10, 21-24
		(b) Give reasons for non-participation at each stage	NA
		(c) Consider use of a flow diagram	24
Descriptive data	14*	(a) Give characteristics of study participants (eg demographic, clinical, social) and information on exposures and potential confounders	21-24
		(b) Indicate number of participants with missing data for each variable of interest	NA
		(c) *Cohort study*—Summarise follow-up time (eg, average and total amount)	NA
Outcome data	15*	*Cohort study*—Report numbers of outcome events or summary measures over time	10-11, 21-24
		Case-control study—Report numbers in each exposure category, or summary measures of exposure	*NA*
		Cross-sectional study—Report numbers of outcome events or summary measures	*NA*
Main results	16	(*a*) Give unadjusted estimates and, if applicable, confounder-adjusted estimates and their precision (eg, 95% confidence interval). Make clear which confounders were adjusted for and why they were included	11
		(*b*) Report category boundaries when continuous variables were categorized	NA
		(*c*) If relevant, consider translating estimates of relative risk into absolute risk for a meaningful time period	NA
Other analyses	17	Report other analyses done—eg analyses of subgroups and interactions, and sensitivity analyses	10, 21-25
**Discussion**			
Key results	18	Summarise key results with reference to study objectives	12
Limitations	19	Discuss limitations of the study, taking into account sources of potential bias or imprecision. Discuss both direction and magnitude of any potential bias	15
Interpretation	20	Give a cautious overall interpretation of results considering objectives, limitations, multiplicity of analyses, results from similar studies, and other relevant evidence	12-16
Generalisability	21	Discuss the generalisability (external validity) of the study results	12-16
**Other information**			
Funding	22	Give the source of funding and the role of the funders for the present study and, if applicable, for the original study on which the present article is based	1

*Give information separately for cases and controls in case-control studies and, if applicable, for exposed and unexposed groups in cohort and cross-sectional studies.

Note: An Explanation and Elaboration article discusses each checklist item and gives methodological background and published examples of transparent reporting. The STROBE checklist is best used in conjunction with this article (freely available on the Web sites of PLoS Medicine at http://www.plosmedicine.org/, Annals of Internal Medicine at http://www.annals.org/, and Epidemiology at http://www.epidem.com/). Information on the STROBE Initiative is available at www.strobe-statement.org.

For peer review only - http://bmjopen.bmj.com/site/about/guidelines.xhtml
For peer review only

BMJ Open

Incidence of Hospitalized Acute Lower Respiratory Tract Disease Including Pneumonia and Lower Respiratory Tract Infection in Bristol, England, 2019

Journal:	*BMJ Open*
Manuscript ID	bmjopen-2021-057464.R1
Article Type:	Original research
Date Submitted by the Author:	29-Mar-2022

Complete List of Authors:	Hyams, Catherine; University of Bristol, Academic Respiratory Unit; University of Bristol, Bristol Vaccine Centre<br>Begier, Elizabeth; Pfizer Inc<br>Garcia Gonzalez, Maria; University of Bristol; Southmead Hospital, Academic Respiratory Unit<br>Southern, Jo; Pfizer Ltd<br>Campling, James; Pfizer Ltd<br>Gray, Sharon; Pfizer Inc<br>Oliver, Jennifer; University of Bristol, Schools of Cellular and Molecular Medicine and Population Health Sciences<br>Gessner, Bradford; Pfizer Inc<br>Finn, Adam; University of Bristol, Bristol Vaccine Centre

**Primary Subject Heading**:	Respiratory medicine
Secondary Subject Heading:	Infectious diseases, Epidemiology
Keywords:	Heart failure < CARDIOLOGY, Respiratory infections < THORACIC MEDICINE, Epidemiology < INFECTIOUS DISEASES

SCHOLARONE™
Manuscripts

I, the Submitting Author has the right to grant and does grant on behalf of all authors of the Work (as defined in the below author licence), an exclusive licence and/or a non-exclusive licence for contributions from authors who are: i) UK Crown employees; ii) where BMJ has agreed a CC-BY licence shall apply, and/or iii) in accordance with the terms applicable for US Federal Government officers or employees acting as part of their official duties; on a worldwide, perpetual, irrevocable, royalty-free basis to BMJ Publishing Group Ltd ("BMJ") its licensees and where the relevant Journal is co-owned by BMJ to the co-owners of the Journal, to publish the Work in this journal and any other BMJ products and to exploit all rights, as set out in our licence.

The Submitting Author accepts and understands that any supply made under these terms is made by BMJ to the Submitting Author unless you are acting as an employee on behalf of your employer or a postgraduate student of an affiliated institution which is paying any applicable article publishing charge ("APC") for Open Access articles. Where the Submitting Author wishes to make the Work available on an Open Access basis (and intends to pay the relevant APC), the terms of reuse of such Open Access shall be governed by a Creative Commons licence – details of these licences and which Creative Commons licence will apply to this Work are set out in our licence referred to above.

Other than as permitted in any relevant BMJ Author's Self Archiving Policies, I confirm this Work has not been accepted for publication elsewhere, is not being considered for publication elsewhere and does not duplicate material already published. I confirm all authors consent to publication of this Work and authorise the granting of this licence.

For peer review only

Title: Incidence of Hospitalized Acute Lower Respiratory Tract Disease Including Pneumonia and Lower Respiratory Tract Infection in Bristol, England, 2019

Authors: Catherine Hyams[1,2,3], Elizabeth Begier[4], Maria Garcia Gonzalez[1,2], Jo Southern[5], James Campling[5], Sharon Gray[4], Jennifer Oliver[1], Bradford D. Gessner[4], Adam Finn[1]

Affiliations:

[1] Bristol Vaccine Centre, Schools of Cellular and Molecular Medicine and Population Health Sciences, University of Bristol, Bristol, BS2 8AE, United Kingdom

[2] Academic Respiratory Unit, Learning and Research Building, Southmead Hospital, Bristol, UK, BS10 5NB

[3] Acute Medical Unit, Southmead Hospital, Bristol, UK, BS10 5NB

[4] Vaccines Medical Development, Scientific and Clinical Affairs, Pfizer Inc, Collegeville, PA, USA

[5] Vaccines Medical Affairs, Pfizer Ltd, Tadworth, UK, KT20 7NS

Corresponding author: Catherine Hyams, MBBS BSc(Hons) PhD
Population Health Sciences, University of Bristol,
Bristol, BS2 8AE,
United Kingdom
catherine.hyams@nbt.nhs.uk
Catherine.hyams@bristol.ac.uk

Alternate author: Maria Garcia Gonzalez
qa18884@bristol.ac.uk

Running Title: Incidence of acute lower respiratory tract disease in adults

Keywords: Pneumonia, respiratory infection, lower respiratory tract infection, heart failure

Funding: CH was funded by the National Institute for Health Research (NIHR) [NIHR Academic Clinical Fellowship (ACF-2015-25-002]. The views expressed are those of the author(s) and not necessarily those of the NIHR or the Department of Health and Social Care. The remainder of the study funding was from Pfizer, Inc (WI255886-1).

For peer review only - http://bmjopen.bmj.com/site/about/guidelines.xhtml

Competing Interests: EB, JS, JC, SG, and BG are full time employees of Pfizer Vaccines and hold stock or stock options. CH is Principal Investigator of the Avon CAP study which is an investigator-led University of Bristol study funded by Pfizer and has previously received support from the NIHR in an Academic Clinical Fellowship. AF is a member of the Joint Committee on Vaccination and Immunization (JCVI) and chair of the World Health Organization European Technical Advisory Group of Experts on Immunization (ETAGE) committee. In addition to receiving funding from Pfizer as Chief Investigator of this study, he leads another project investigating transmission of respiratory bacteria in families jointly funded by Pfizer and the Gates Foundation. The other authors have no relevant conflicts of interest to declare.

Word count: **2997/4000**

Summary (38/40 words): Using both retrospective and prospective methodologies, we found a high annual incidence of acute lower respiratory disease (>1700 per 100000; 1.7%) in and around Bristol, UK: pneumonia (~0.6%), lower respiratory tract infection without pneumonia (>0.7%), and heart failure (>0.3%).

Author Contributions: CH, EB, MGG, JS, BDG and AF generated the research questions and analysis plan CH, EB, MGG, JS, JC, SG and BDG undertook and contributed to the data analysis. AF oversaw the research and data collection which was undertaken by CH and MGG. All authors contributed to the preparation of the manuscript.

For peer review only - http://bmjopen.bmj.com/site/about/guidelines.xhtml

Abstract (278/300):

Objectives

To determine the disease burden of acute lower respiratory tract disease (aLRTD) and its subsets (pneumonia, lower respiratory tract infection [LRTI], heart failure) in hospitalized adults in Bristol, UK.

Setting

Single-centre, secondary care hospital, Bristol UK

Design

We estimated aLRTD hospitalizations incidence in adults (≥18 years) in Bristol, UK using two approaches. First, retrospective ICD-10 code analysis (first five positions/hospitalization) identified aLRTD events over a 12-month period (March2018–Feb2019). Secondly, during a 21-day prospective review (19Aug–9Sept2019), aLRTD admissions were identified, categorized by diagnosis, and subsequently annualized. Hospital catchment denominators were calculated using linked general practice and hospitalization data, with each practice's denominator contribution calculated based on practice population and percent of the practices' hospitalizations admitted to the study hospital.

Participants

Prospective review: 1322 adults screened; 410 identified with aLRTD. Retrospective review: 7727 adult admissions.

Primary and Secondary outcome measures

The incidence of aLRTD and its subsets in the adult population of Southmead Hospital, Bristol UK.

Results

Based on ICD-10 code analysis, annual incidences per 100000 population were: aLRTD, 1901; pneumonia, 591; LRTI, 739; heart failure, 402. aLRTD incidence was highest among those ≥65-years: 65–74 (3684 per 100000 adults), 75–84 (6962 per 100000 adults), and ≥85 (11430 per 100000 adults).

For peer review only - http://bmjopen.bmj.com/site/about/guidelines.xhtml

During the prospective review, 410/1322 (31%) hospitalized adults had aLRTD signs/symptoms, and annualized incidences closely replicated retrospective analysis results.

Conclusions

aLRTD disease burden was high, increasing sharply with age. aLRTD incidence is probably higher than estimated previously due to criteria specifying respiratory-specific symptoms or radiological change, usage of only the first diagnosis code, and mismatch between case count sources and population denominators. This may have significant consequences for healthcare planning, including usage of current and future vaccinations against respiratory infection.

Strengths and Limitations of This Study

- We used two analytical methods at the same site over a comparable period, to calculate incidence using both prospective and retrospective approaches.
- The case burden of aLRTD and its subgroups was pre-defined and included patients with atypical presentations
- We calculated incidence using a denominator derived from GP records, providing increased accuracy compared to population calculations based on census data.
- This was a single-center study, with a predominantly Caucasian cohort; therefore, the findings might not be generalizable to other populations.
- The ICD-10 coding data analysis was limited to codes within the first five positions, and therefore may have excluded some cases where other diagnoses were placed higher in the diagnostic coding hierarchy.

For peer review only - http://bmjopen.bmj.com/site/about/guidelines.xhtml

Introduction

Acute lower respiratory tract disease (aLRTD) encompasses pneumonia, non-pneumonic lower respiratory tract infection (NP-LRTI), acute bronchitis, exacerbation of underlying respiratory diseases (including asthma and chronic obstructive pulmonary disease [COPD]), and acute heart failure (HF) events resulting in respiratory symptoms (e.g. breathlessness). Before the COVID-19 pandemic, European healthcare costs for pneumonia alone in adults were estimated at €10 billion annually, including €5.7 billion for inpatient care. [1] Pneumonia incidence in Europe varies by country and intra-country region, age, socioeconomic status, and gender; [2-4] however, in all studies pneumonia incidence in adults increases sharply with age. [3] Pneumonia affects an estimated 0.5% to 1% of UK adults each year [5, 6]. Overall LRTI incidence is considerably higher with ~15% of UK adults aged ≥65 years experiencing an event each year.[7] Whilst HF is not typically clinically included as an acute respiratory illness, HF with respiratory symptoms may be caused by respiratory viral infection, such as respiratory syncytial virus (RSV), either acutely or 3-4 weeks after the primary infection [8, 9].

However, aLRTD incidence may be considerably higher than previously reported, given that published literature has documented several reasons why previous estimates may have been erroneously low [1]. Estimates of aLRTD based on pneumonia defined as radiologically demonstrated alveolar infiltrates, may underestimate true disease burden as chest radiography (CXR) is an imperfect gold-standard. [10, 11] Immunosuppressed, elderly or dehydrated patients are likely to be under-represented if respiratory infection is defined by radiologically demonstrated changes. [10, 11] Microbiological investigations for pneumonia are undertaken variably and identify a causative pathogen in 50% of cases at most [12, 13]; hence, the disease is probably under-reported when confirmed microbiological diagnosis is required. Furthermore, Respiratory Syncytial Virus (RSV) infection has recently been recognized as an important respiratory pathogen later in life, [9] with severe disease occurring in patient groups in whom the diagnosis is likely to be under-recognized (e.g.

For peer review only

the elderly or those with underlying cardiac conditions). [8] Studies of clinical coding data are retrospective and subject to recognized limitations associated with this methodology [14, 15]. Older patients with pneumonia often have atypical presenting signs and symptoms, which may lead to missed or incorrect admission diagnoses. [16] Pneumonia may occur secondary to, or be an underlying cause of, the main presenting complaint, particularly in patients with cerebrovascular accidents (CVA), HF, COPD exacerbations or altered consciousness levels. [17] In these scenarios, pneumonia may not be the primary hospitalization diagnosis code and may not even be coded as an associated diagnosis.

There are many studies examining the incidence of acute respiratory illness in children; however, data on respiratory illness in adults in the UK is lacking. Given the paucity of data supporting accurate aLRTD incidence rates and its disease subsets in adults, we undertook to assess aLRTD incidence by two approaches (retrospective and prospective) in Bristol, UK, seeking to determine the disease burden of hospitalized aLRTD and its subgroups more accurately.

For peer review only

For peer review only - http://bmjopen.bmj.com/site/about/guidelines.xhtml

Methods

Study Design

This study was conducted at a large secondary care institution in UK (North Bristol NHS Trust) with specialist Respiratory Services (interstitial lung disease, pleural disease). Two approaches were undertaken to estimate aLRTD incidence: (1) “retrospective analysis” of aLRTD International Classification of Diseases Tenth Revision (ICD-10) diagnostic codes for an entire year; and, (2) 21-day observational “prospective review” of aLRTD hospital admissions.

Ethics

This study was approved by North Bristol NHS Trust Research Audit Ethics Committee (CA52218).

Patient and Public Involvement

No patient involved.

Retrospective Analysis

For the retrospective analysis, all adult inpatient admissions (≥18 years) obtained from Hospital Episode Statistic (HES) to the study hospital during March 2018–February 2019 with aLRTD ICD-10 diagnostic codes (Supplementary data 1) in any of the first 5 positions were identified and categorized into aLRTD subgroups: pneumonia, NP-LRTI, other LRTD, and HF (Supplementary data 2). A mutually exclusive hierarchy was used (pneumonia, NP-LRTI, then other LRTD) although HF diagnoses could co-occur with other categories. “Other LRTD” included acute respiratory events that could not definitively be placed in another category. Only the first 5 ICD-10 codes were available for analysis.

Prospective Review

Adult patients (≥18 years) resident within Bristol, North Somerset, and South Gloucestershire Clinical Commissioning Group (CCG) referred to the Acute Medical Unit (AMU) at North Bristol NHS Trust during 19th August –9th September 2019 were included in an audit on acute respiratory illness. This

For peer review only - http://bmjopen.bmj.com/site/about/guidelines.xhtml

time period was selected because it was felt to represent a period when there were an average number of adults hospitalised with aLRTD. A respiratory physician (CH) reviewed presenting features and investigation results for each admitted patient to determine whether aLRTD was present. Further medical record review was undertaken if patients had: new/worsening breathlessness, cough or sputum production; new or worsening peripheral oedema; pleurisy; clinical examination findings consistent with respiratory infection or HF; or fever attributable to suspected respiratory infection. Patients with non-respiratory diagnoses were excluded. There were no patient refusals for either approach.

Prospective Review Outcome measures

aLRTD was considered confirmed in individuals with: new/worsening respiratory symptoms, with or without fever; sepsis, delirium or raised inflammatory markers attributable to likely respiratory infection in admitting clinical team's opinion; radiological change in keeping with infection (e.g. consolidation); and/or final diagnosis of NP-LRTI, pneumonia or infective exacerbation of a chronic respiratory condition. A pneumonia diagnosis was assigned if radiological changes likely due to infection were described by the reporting radiologist. An NP-LRTI diagnosis was assigned if aLRTD signs and symptoms likely to be due to infection were present without demonstrated radiological change. A HF diagnosis was assigned in presence of: new/worsening breathlessness and bi-basal crepitations, cardiac wheeze or new/worsening bilateral pitting oedema; elevated pro-NT BNP (≥450pg/mL); radiologist-reported radiographic changes consistent with cardiac failure; or a final consultant physician diagnosis of HF, cardiac failure or left-ventricular failure. When present, ≥1 diagnosis was selected.

For both retrospective and prospective studies, pneumonia included both community and healthcare setting acquired cases; although, the prospective review only captured admitting diagnoses and pneumonias occurring later during hospitalization were not included.

Incidence calculations

For peer review only - http://bmjopen.bmj.com/site/about/guidelines.xhtml

Annual incidence per 100000 persons was calculated for both retrospective and prospective studies. Case counts from prospective review were annualized (i.e., case counts by diagnosis and overall were divided by the percentage of annual admissions for these diagnosis groups that occurred in this 21-day period in the retrospective analysis).

Incidence Denominators

To calculate appropriate population denominators for incidence calculations, aLRTD hospital admission event data were linked to aggregated GP practice patient registration data within the NHS Bristol, North Somerset, and South Gloucestershire CCG for 2017–2019. Only 4% of patients sought care at North Bristol NHS Trust hospital from outside these local CCGs, despite presence of specialist respiratory services. In the UK, GP registration is available free of charge for all, regardless of residential status. For GP practices within these same CCGs, the proportion of their aLRTD admissions occurring at North Bristol NHS Trust was multiplied by their patient registration count in 2019 by age group, to get each practice's contribution to the denominator (e.g. if 50% aLRTD admissions were at North Bristol among persons 50−64 years, the practice would contribute half of their patients 50–64 years to the denominator). Further details of this methodology have been described previously. [18]

Statistical analysis

Patient characteristics were tabulated by aLRTD diagnosis. Categorical variables were presented as counts with percentages. Continuous data are presented with means and standard deviations (SD) if normally distributed and medians and interquartile range (IQR) if not normally distributed. Patient groups difference were evaluated using the Friedman test with Wilcoxon signed-rank test.

For peer review only

Results

Retrospective analysis

Over a 12-month period, we identified 7727 hospital admissions for aLRTD: 3005 NP-LRTI admissions, 2402 pneumonia, 1633 HF and 1071 other LRTD (Table 1). aLRTD admissions were lowest in March and April and highest December through February (Figure 1A), overall and for all aLRTD subgroups (P<0.05) (Figure 1B-D). Overall, 28.1% (2244) cases were identified as being potentially hospital-acquired infection based on co-occurring ICD-10 code 'Y95 Nosocomial Infection'.

Prospective review

Among 1322 eligible adult patients referred to AMU over the 21-day review period (Figure 2), 410 patients had signs or symptoms of aLRTD: 188 (46%) NP-LRTI; 152 (37%) pneumonia, and 77 (19%) HF. Seven patients had both decompensated HF and a respiratory infection at hospital admission. On admission, >10% of aLRTD patients did not have respiratory symptoms: 16 (11%) pneumonia, 25 (13%) NP-LRTI, and 18 (14%) HF (Table 2).

Almost all adults admitted with aLRTD underwent routine biochemistry, hematology, and radiological investigation (99.9%, n=409). In contrast, only 150 (37%) patients with aLRTD had microbiological testing performed: blood cultures (n=149, 36%) and urine cultures (n=143, 35%). Pneumonia patients more commonly underwent microbiological investigation than NP-LRTI patients (P<0.05) with highest disparity in rates of sputum culture, urinary antigens, and respiratory viral PCR (Table 2). All cardiac failure patients who underwent microbiological investigation had concomitant respiratory infection (Table 2). Overall, a microbiological diagnosis was found in 11 (3%) cases, highlighting the low frequency of definitive pathogen identification in aLRTD. Younger patients underwent microbiological testing more frequently than the elderly for all aLRTD categories (Table 2).

For peer review only - http://bmjopen.bmj.com/site/about/guidelines.xhtml

For peer review only

Disease incidence

Retrospective analysis yielded an overall aLRTD incidence of 1901 per 100000. Disease incidence rose with increasing age (Table 3), both overall and for all disease subgroups; incidences per 100000 among adults aged ≥85 years were: 11430 (aLRTD), 6116 (NP-LRTI), 4215 (pneumonia) and 4005 (HF). Overall, 28.1% aLRTD hospitalizations also included an ICD-10 discharge code for 'nosocomial infection', suggesting the aLRTD event or other infection was hospital-acquired. For pneumonia 25.3% had the nosocomial infection code. If all of these were related to the pneumonia diagnosis, an estimated residual 1794 events would have been community-acquired pneumonia (annual incidence 441/100000 adults). Among older age categories, where the incidence of aLRTD was highest, NP-LRTI incidence was similar to pneumonia incidence, with an approximate 1:1 ratio of NP-LRTI to pneumonia cases. However, among adults under age 50 years, there were approximately twice as many NP-LRTI cases observed as pneumonia cases. Incidence calculations using annualized prospective review results were broadly comparable with retrospective analysis of ICD-10 data (Table 3).

For peer review only

For peer review only - http://bmjopen.bmj.com/site/about/guidelines.xhtml

Discussion:

This is the first UK study to assess aLRTD incidence comprehensively. We conducted an analysis of 12-months of hospital admissions by ICD-10 diagnosis code data and a 21-day prospective review at a large academic hospital in southwest England. With both approaches, we found a high annual incidence of aLRTD (>1700 per 100000; 1.7%), pneumonia (~0.6%), NP-LRTI without pneumonia (>0.7%), and HF (>0.4%). Incidences increased sharply in a non-linear manner as age increased above 65 years for all aLRTD categories. These results suggest rates are probably significantly higher than previous disease estimates from the UK (Table 4) but comparable with many results globally, [19, 20] with important consequences for healthcare resources. For example, a recent review highlighted that pneumonia incidences ranged from 1000 to 2500 per 100000 (1–2.5%) among persons aged 65–74 years in Spain, Germany, France, Japan and the US, which are comparable to the >1250 per 100000 (1.3%) reported here. Some of the potential sources of underestimation for other UK incidence studies (Table 4) include: case source and denominator mismatch; use of first position in ICD-code analysis only; inclusion of enrolled subjects in consent-based prospective studies only; requiring specific symptoms and chest x-ray confirmation in order to be classified as pneumonia/aLRTD; and, lastly, the rising incidence of aLRTD.

For peer review only

Comparison with published literature

No studies have reported aLRTD incidence comprehensively in UK hospitalized patients within the last 20 years. However, eight publications report incidence of ≥1 aLRTD subgroup. Seven publications reported community-acquired pneumonia (CAP) incidence (three from Nottingham, UK). For pneumonia, our incidence estimates were three to four-fold higher than other UK inpatient incidence estimates (Table 4) but comparable to estimates from other countries. [19, 20] Only two UK studies from approximately 20 years ago reported NP-LRTI incidence (one with both CAP and NP-LRTIs; Table 4), and only one provided an inpatient estimate. [21] NP-LRTI incidence was approximately 2-fold

For peer review only - http://bmjopen.bmj.com/site/about/guidelines.xhtml

lower than that calculated here, taking into account inclusion of CAP and other NP-LRTI in their estimates. [21, 22] The one UK study reporting HF incidence had methodological differences (i.e., inclusion of outpatients and limiting to initial HF diagnosis) and estimates could not be compared. [23] Close examination of the existing literature methods yielded multiple sources for potential underestimation.

First, for incidence studies that were not countrywide, identifying an appropriate denominator is challenging. Like many other inpatient settings worldwide, UK hospitals' catchment areas for acute treatment are principally driven by geography, but the proportion of any area's residents expected to use the hospital becomes less clear as distance from the hospital increases because catchment areas and populations of different hospitals may overlap. Defining hospital catchment populations based solely on census data cannot account for this variability. Including all geographic areas using the hospital to any extent results in population denominator overestimation and underestimated incidence. Here, we addressed this by calculating population denominators based on hospital utilization behavior from referring General Practices.

Second, other studies used fewer codes in their ICD code analysis: all limited their analyses to events where the diagnostic code was in the first position (Table 4; case definition column), potentially excluding admissions in which pneumonia/NP-LRTI complicated other underlying respiratory diseases, including COPD and asthma. Limiting to first position has been shown to reduce sensitivity for pneumonia events by about 30% (66%–72% sensitive). [22, 24] Conversely, the recent British Thoracic Society (BTS) audit on CAP found ~27% of pneumonia events identified by ICD code (J12-18) had no new CXR infiltrates. [6] Even accounting for this potential over coding practice, our estimates remain well above other published UK estimates.

Third, for other prospective studies, exclusion of events where patients did not consent to participation or were not identified by study surveillance processes (often conducted predominately during business hours) can introduce underestimation. Further, other prospective pneumonia studies

For peer review only - http://bmjopen.bmj.com/site/about/guidelines.xhtml

specifically required documentation of specific symptoms, radiological findings, and treatments, [25] potentially excluding those without these features documented in medical records. In our prospective review, approximately 11% did not display typical signs and symptoms of pneumonia and could have been excluded by that requirement. Requiring CXR confirmation has been shown to reduce incidence estimates for pneumonia, [20] although all pneumonia events in our prospective review were radiologically confirmed.

Fourth, trends over time may also contribute to our estimates being higher than previous reports. Our study's estimates are recent, and rising incidence of pneumonia has been documented in all studies that have reported such trends. [25-27]

Finally, this study included, in part, hospital-acquired pneumonias (HAP), which were excluded from estimates calculated in some other studies (Table 4). The retrospective analysis may have included more nosocomial infection than the prospective review, as the latter was focused on evaluation of patients at admission for aLRTD and would not have reliably captured events that developed during hospitalization. 25.3% pneumonia events included a nosocomial infection code, but this code could relate to any nosocomial infection during that hospitalization. If all these cases were assumed to be hospital-acquired pneumonia, our estimates community-acquired pneumonia incidence would still be well above prior UK estimates: 441/100000 (≥18 years).

While not impacting all-cause aLRTD incidence estimates discussed above, during the prospective review, we found low rates of microbial investigation which prevented us from generating pathogen-specific incidence estimates. Only 52% of patients with radiologically-confirmed pneumonia underwent microbiological testing during hospitalization, with even lower rates in other aLRTD subgroups (41% NP-LRTI and 14% HF). Microbiological testing occurred less frequently as age increased, particularly in NP-LRTI patients. It is possible that, because aLRTD hospitalizations are substantially more common among older persons, less etiologic investigation is performed. Furthermore, clinicians may elect to treat elderly patients with a more pragmatic and less invasive

For peer review only - http://bmjopen.bmj.com/site/about/guidelines.xhtml

approach. Management guidelines do not require specific pathogen identification to inform treatment choice. Presence only of atypical features on presentation (in this series, 13% NP-LRTI and 11% pneumonia cases) may also reduce the likelihood of timely microbiological testing. Low rates of microbiological testing, and consequently of confirmed microbiological diagnosis, may represent a source of underestimation of pathogen-specific disease incidence in patient groups (i.e., testing bias), particularly in elderly patient groups.

Strengths and Limitations of This Study

This study has many strengths. First, this study used two analytical methods at the same site over a comparable period, to calculate incidence using both prospective and retrospective approaches. Second, the case burden of aLRTD and its subgroups was pre-defined and included patients with atypical presentations but with clinical and/or radiological diagnoses, who may otherwise have been excluded from analysis. Additionally, we calculated incidence using a denominator derived from GP records, providing increased accuracy compared to population calculations based on census data.

However, the study also had some limitations. This was a single-center study, with a predominantly Caucasian cohort; therefore, the findings might not be generalizable to other populations both within the UK and in other countries. Different healthcare systems may affect patient treatment preference, and as the NHS provides care which is free at the point of access, the hospitalization rates seen in this study may be different in fee or insurance based healthcare system. Similarly, physician treatment preferences may affect hospitalization rates, and we have not explored these in this analysis. The ICD-10 coding data analysis was limited to codes within the first five positions, and therefore may have excluded some cases where other diagnoses were placed higher in the diagnostic coding hierarchy. Furthermore, we could not determine how many cases of the 28.1% ICD-10 cases also coded with nosocomial infection had hospital-acquired respiratory infection rather than other nosocomial infections.

For peer review only - http://bmjopen.bmj.com/site/about/guidelines.xhtml

Although the denominator used to calculate incidence was derived from GP records, this was still an estimate as there is no precisely defined denominator hospital catchment. We were unable to exclude patients from outside the local CCGs in the retrospective analysis, due to the way ICD-10 data was obtained. However, these patients were excluded from the prospective review and the incidence calculated was comparable, suggesting any effect that patients attending North Bristol NHS Trust from outside the local CCGs have on incidence estimates is minimal. This may be because any effect of travelling or health-seeking behavior is bi-directional: whilst some patients admitted to Southmead hospital were from outside the local area, it is also true that patients with aLRTD within the relevant CCGs would have been admitted to other hospitals. We also acknowledge the 21-day prospective review period was relatively short, not repeated and may not be fully representative of clinical practice and cases throughout the year. This study was conducted before the emergence of COVID-19, and we think these data will be useful in one of two ways in the context of COVID-19: (1) either COVID-19 will become endemic, and the data will reflect the first year before a new normal, or (2) COVID-19 will abate and it will provide an anchor for understanding incidence during a respiratory viral pandemic'

In conclusion, we found similarly high estimates of LRTD incidence using two different approaches, and these estimates were higher than those obtained previously in the UK. Determining if there is a real increase in incidence, or if this estimate is larger due to more accurate methodology including a more accurate denominator will require ongoing comprehensive surveillance. Nonetheless, combining all types of LRTD highlights the high burden for this important and potentially life-threatening disease group. Incidence assessments require close assessments of potential areas of under ascertainment, including unidentified or unenrolled cases in prospective studies, reduced positions or number of ICD-10 codes included for retrospective studies, and population denominator mismatch for all study types. Our prospective review findings highlight the need to consider atypical clinical presentations for pneumonia and the lack of routine microbiological investigation in many

For peer review only - http://bmjopen.bmj.com/site/about/guidelines.xhtml

aLRTD patients required for pathogen-specific aLRTD incidence calculation. Future research should include a fully prospective assessment of aLRTD incidence with comprehensive diagnostic testing across multiple respiratory seasons, to ensure accurate capture of all aLRTD events, particularly in the elderly. Such research should be undertaken given the high and rising aLRTD burden to enable appropriate healthcare planning and identification of interventions which may reduce disease burden.

For peer review only

For peer review only - http://bmjopen.bmj.com/site/about/guidelines.xhtml

Acknowledgements

We would like to acknowledge the assistance of Qi Yan, PhD (Pfizer, Inc.) who provided indispensable medical writing and literature review support for this manuscript and Harvey Walsh Health who performed the hospital denominator calculation used here. For the denominator analysis, Hospital Episode Statistics (HES) Data were re-used with the permission of NHS Digital via Harvey Walsh Limited.

Data Sharing

No additional data available

For peer review only

For peer review only - http://bmjopen.bmj.com/site/about/guidelines.xhtml

References

1. Gibson, G.J., et al., *Respiratory health and disease in Europe: the new European Lung White Book.* Eur Respir J, 2013. **42**(3): p. 559-63.
2. Cilloniz, C., et al., *Impact of age and comorbidity on cause and outcome in community-acquired pneumonia.* Chest, 2013. **144**(3): p. 999-1007.
3. Torres, A., et al., *Burden of pneumococcal community-acquired pneumonia in adults across Europe: A literature review.* Respir Med, 2018. **137**: p. 6-13.
4. *European Lung White Book. Pneumonia.* April 25, 2021]; Available from: https://www.erswhitebook.org/chapters/acute-lower-respiratory-infections/pneumonia/#:~:text=pneumonia%20(VAP).-,Incidence,000%20cases%20are%20expected%20annually.
5. *British Lung Foundation. Pneumonia Statistics.* April 25, 2021]; Available from: https://statistics.blf.org.uk/pneumonia.
6. Lim, W. and H. Lawrence, *British Thoracic Society. National Audit Report: Adult Community Acquired Pneumonia Audit 2018-2019. National Audit Period: 1 December 2018 – 31 January 2019*. 2019.
7. Millett, E.R.C., et al., *Incidence of community-acquired lower respiratory tract infections and pneumonia among older adults in the United Kingdom: a population-based study.* PloS one, 2013. **8**(9): p. e75131-e75131.
8. Branche, A.R. and A.R. Falsey, *Respiratory Syncytial Virus Infection in Older Adults: An Under-Recognized Problem.* Drugs & Aging, 2015. **32**(4): p. 261-269.
9. Falsey, A.R. and E.E. Walsh, *Respiratory syncytial virus infection in elderly adults.* Drugs & aging, 2005. **22**(7): p. 577-587.
10. Maughan, B.C., et al., *False-negative chest radiographs in emergency department diagnosis of pneumonia.* R I Med J (2013), 2014. **97**(8): p. 20-3.
11. Self, W.H., et al., *High discordance of chest x-ray and computed tomography for detection of pulmonary opacities in ED patients: implications for diagnosing pneumonia.* Am J Emerg Med, 2013. **31**(2): p. 401-5.
12. Cilloniz, C., et al., *Microbial aetiology of community-acquired pneumonia and its relation to severity.* Thorax, 2011. **66**(4): p. 340-6.
13. Jain, S., et al., *Community-Acquired Pneumonia Requiring Hospitalization.* N Engl J Med, 2015. **373**(24): p. 2382.
14. Mainor, A.J., et al., *ICD-10 Coding Will Challenge Researchers: Caution and Collaboration may Reduce Measurement Error and Improve Comparability Over Time.* Med Care, 2019. **57**(7): p. e42-e46.
15. Burns, E.M., et al., *Systematic review of discharge coding accuracy.* J Public Health (Oxf), 2012. **34**(1): p. 138-48.
16. Henig, O. and K.S. Kaye, *Bacterial Pneumonia in Older Adults.* Infect Dis Clin North Am, 2017. **31**(4): p. 689-713.
17. Fine, M.J., et al., *Prognosis and outcomes of patients with community-acquired pneumonia. A meta-analysis.* JAMA, 1996. **275**(2): p. 134-41.
18. Campling, J., et al., *A novel approach to calculate disease incidence for hospital-based health events in England.* The Lancet, 2021. **398**: p. S30.
19. Chalmers, J., et al., *Community-acquired pneumonia in the United Kingdom: a call to action.* Pneumonia (Nathan), 2017. **9**: p. 15.
20. Shi, T., et al., *Global and Regional Burden of Hospital Admissions for Pneumonia in Older Adults: A Systematic Review and Meta-Analysis.* J Infect Dis, 2020. **222**(Suppl 7): p. S570-S576.
21. Lovering, A.M., et al., *Epidemiology and resource utilization for patients hospitalized for lower respiratory tract infection.* Clin Microbiol Infect, 2001. **7**(12): p. 666-70.

For peer review only

For peer review only - http://bmjopen.bmj.com/site/about/guidelines.xhtml

22. Yu, O., et al., *Classification algorithms to improve the accuracy of identifying patients hospitalized with community-acquired pneumonia using administrative data.* Epidemiology and Infection, 2011. **139**(9): p. 1296-1306.
23. Uijl, A., et al., *Risk factors for incident heart failure in age- and sex-specific strata: a population-based cohort using linked electronic health records.* Eur J Heart Fail, 2019. **21**(10): p. 1197-1206.
24. van de Garde, E.M.W., et al., *International classification of diseases codes showed modest sensitivity for detecting community-acquired pneumonia.* Journal of Clinical Epidemiology, 2007. **60**(8): p. 834-838.
25. Pick, H., et al., *Pneumococcal serotype trends, surveillance and risk factors in UK adult pneumonia, 2013–18.* Thorax, 2020. **75**(1): p. 38.
26. Elston, J.W.T., et al., *Increasing incidence of invasive pneumococcal disease and pneumonia despite improved vaccination uptake: surveillance in Hull and East Yorkshire, UK, 2002–2009.* Epidemiology and Infection, 2012. **140**(7): p. 1252-1266.
27. Thorrington, D., et al., *Elucidating the impact of the pneumococcal conjugate vaccine programme on pneumonia, sepsis and otitis media hospital admissions in England using a composite control.* BMC Medicine, 2018. **16**(1): p. 13.

For peer review only

For peer review only - http://bmjopen.bmj.com/site/about/guidelines.xhtml

Table 1: Demographic Characteristics of Patients admitted with aLRTD for 1 Year ICD-10 code Retrospective Analysis and 21-day Prospective Review Period -- 2018–2019

Characteristic	Pneumonia		NP-LRTI		Heart Failure		Other LRTD	All LRTD	
Study	Prospective review	Retrospective analysis	Prospective review	Retrospective analysis	Prospective review	Retrospective analysis	Retrospective Review Only	Prospective review	Retrospective analysis
N	152	2402	188	3005	77	1633	1071	410	7727
Gender, females	61 (40)	1078 (45)	99 (53)	1482 (49)	39 (51)	731 (45)	489 (46)	194 (47)	3780 (49)
**Age**									
Median (IQR), y	80 (67-86)	81 (66-88)	70 (46-87)	69 (45-87)	87 (72-90)	87 (70-90)	74 (53-82)	80 (64-88)	81 (65-90)
18-24	4 (3)	72 (3)	9 (5)	151 (5)	0 (0)	0 (0)	26 (2)	13 (3)	249 (3)
25-34	2 (2)	48 (2)	12 (6)	183 (6)	0 (0)	3 (0)	33 (3)	14 (3)	267 (3)
35-44	6 (4)	97 (4)	14 (7)	209 (7)	2 (3)	10 (1)	59 (6)	22 (5)	375 (5)
45-54	8 (5)	118 (5)	11 (6)	183 (6)	0 (0)	22 (1)	112 (10)	19 (5)	435 (6)
55-64	18 (12)	293 (12)	19 (10)	305 (10)	8 (10)	158 (10)	210 (20)	45 (11)	966 (13)
65-74	34 (22)	501 (21)	32 (17)	549 (18)	10 (15)	199 (12)	223 (21)	75 (18)	1472 (19)
75-84	44 (28)	667 (28)	40 (21)	621 (21)	20 (30)	498 (31)	205 (19)	100 (24)	1991 (26)
≥85	38 (26)	606 (25)	51 (27)	704 (23)	37 (55)	742 (45)	203 (19)	123 (30)	2255 (29)

For peer review only - http://bmjopen.bmj.com/site/about/guidelines.xhtml

Table 2. Clinical Characteristics and Investigations of Patients admitted with aLRTD over 21-day Prospective Review Period in August-September 2020

Characteristic	Pneumonia n=152 (%)	NP-LRTI n=188 (%)	Heart Failure n=77 (%)	All LRTD n=410 (%)
GP	56 (37)	72 (39)	30 (39)	158 (39)
A&E department	93 (61)	100 (54)	45 (58)	238 (58)
Transfer from another unit	2 (1)	13 (7)	0 (0)	15 (4)
Other	1 (1)	1 (1)	2 (3)	4 (1)
**Referral Source**				
Typical features†	136 (89)	163 (87)	63 (82)	355 (87)
Atypical features	16 (11)	25 (13)	14 (18)	55 (13)
- collapse/falls	11 (7)	12 (6)	0 (0)	23 (6)
- confusion	0 (0)	7 (4)	4 (5)	10 (2)
- drowsiness	1 (1)	1 (1)	2 (3)	4 (1)
- off legs/generally unwell	5 (3)	5 (3)	8 (10)	18 (4)
**LRTD Signs and symptoms on referral to AMU**				
Biochemistry	152 (100)	185 (99)	77 (100)	419 (100)
Haematology	152 (100)	185 (99)	77 (100)	419 (100)
Radiology	152 (100)	185 (99)	77 (100)	419 (100)
**Investigations Performed**				
Testing by Age Group				
All patients	79/152 (52)*	77/188 (41)	11/77 (14)	167 (41)
18-24	3/4 (75)	6/9 (66)	0/0 (0)	9/13 (69)
25-34	0/0 (0)	6/12 (50)	0/0 (0)	6/12 (50)
35-44	5/6 (83)	10/14 (71)	2/2 (100)	17/22 (77)
45-54	6/8 (75)	6/11 (55)	0/0 (0)	13/19 (68)
55-64	11/18 (61)	12/19 (63)	5/8 (63)	31/45 (69)
65-74	15/34 (44)	12/32 (38)	1/10 (10)	28/75 (37)
75-84	21/43 (49)	10/40 (25)	2/20 (10)	33/100 (33)
≥85	18/39 (46)	15/51 (19)	1/37 (3)	34/124 (27)
Test performed				
Blood culture	79 (52)	70 (37)	5 (6)	150 (37)
Urine culture	66 (43)	77 (41)	11 (14)	150 (37)
Sputum culture	27 (18)*	7 (4)	2 (3)	35 (9)
BinaxNOW® Pn UAT ‡	29 (19)*	6 (3)	0 (0)	35 (9)
Respiratory virus PCR	16 (11)*	11 (6)	1 (1)	28 (7)
Pleural fluid culture	3 (2)	0 (0)	1 (1)	4 (1)

A&E, Accident and Emergency department; GP, general practitioner; LRTD, lower respiratory tract disease; NP-LRTI, non-pneumonic lower respiratory tract infection; IQR, interquartile range; Pn UAT, pneumococcal urinary antigen test; PCR, polymerase chain reaction.
* $P<0.05$.
† Typical symptoms included cough, breathlessness, increased or discolored sputum production, wheeze, pleurisy, peripheral oedema, hemoptysis, reduced exercise tolerance and/or fever.
‡ BinaxNOW Pn UAT was only performed in accordance with NICE/BTS guidelines.

For peer review only - http://bmjopen.bmj.com/site/about/guidelines.xhtml

Table 3: Incidence of aLRTD resulting in hospital admission based on prospective and retrospective approaches by age group and condition, North Bristol National Health Service Trust— United Kingdom 2018–2019.

	Age Groups					
	**All Adults**	**18-49 y**	**50-64 y**	**65-74 y**	**75-84 y**	**≥85 y**
**Population in 2018**	406481	226920	91534	45705	29487	12835
**Retrospective Analysis of a Year's ICD-10 codes**						
**Annual cases – N (row %)**						
All aLRTD	7,727	1,130 (14)	1,103 (14)	1,684 (22)	2,053 (27)	1,757 (23)
Pneumonia	2,402	264 (11)	288 (12)	589 (25)	720 (30)	541 (22)
NP-LRTI	3,005	576 (19)	410 (14)	572 (19)	662 (22)	785 (26)
Other LRTD	1,071	246 (23)	268 (25)	226 (21)	200 (19)	131 (12)
Heart Failure	1,633	48 (3)	189 (12)	397 (24)	485 (30)	514 (31)
**LRTI/Pneumonia Ratio**	1.3	2.2	1.4	1.0	0.9	1.5
**Incidence (per 100,000)**						
All aLRTD	1,901	497	1,205	3,684	6,962	13,689
Pneumonia	591	116	315	1,289	2,442	4,215
NP-LRTI	739	254	448	1,252	2,245	6,116
Other LRTD	263	108	293	494	678	1,021
Heart Failure	402	21	206	869	1,645	4,005
**21-day Prospective Review (annualized)**						
**Annualized cases – N (row %)**						
All aLRTD	7,885	1,038	962	1,692	2,231	1,962
Pneumonia	2,621	224	397	776	690	534
NP-LRTI	3,857	796	531	653	1,061	816
Heart Failure	2,000	51	205	308	641	795
**LRTI/Pneumonia Ratio**	1.5	3.6	1.3	0.8	1.5	1.5
**Incidence (per 100,000)**						
All aLRTD	1,940	458	1,050	3,703	7,565	15,283
Pneumonia	645	99	433	1,698	2,339	4,164
NP-LRTI	944	351	580	1,429	3,599	6,360
Heart Failure	492	23	224	673	2,174	6,193

LRTI = lower respiratory tract infection; LRTD = lower respiratory tract disease.
Pneumonia category includes community and healthcare acquired. For retrospective ICD-10 based cohort, the following mutually exclusive hierarchy was used to define pneumonia, LRTI, and other LRTD; heart failure event could overlap with other categories. "Other LRTD" contains LRTD events that could not definitively be placed in one of the other respiratory disease categories.

For peer review only - http://bmjopen.bmj.com/site/about/guidelines.xhtml

For peer review only

Table 4: Literature review of aLRTD incidence in hospitalized adults, United Kingdom

Study	Study Years	Location (Facility)	Event Setting	Age	Case Definition[b]	Key inclusion	Denominator Source	Overall Incidence	Age Breakdown (years)	Incidence per 100,000 by age[d]	Comments
**Community –acquired Pneumonia**											
Current Study	2018 – 2019	Bristol (Southmead Hospital)	Inpatients only	≥18 y	Clinical signs/symptoms with radiological change in keeping with infection (prospective review portion) **AND** Retrospective ICD-10 code analysis (1st 5 positions): J12-J18, J85, and J86	Hospital-acquired pneumonia (HAP) included	Based on number of persons ≥18 years registered in referring GP practices. For practices with split referral patterns, number adjusted for percent of admissions that came to Southmead.	648	18 –49	116	Retrospective analysis includes 1st 5 positions
									50 –64	315	
									65 –74	1,289	
								591	75 –84	2,442	
									≥85	4,215	
Elston 2012, Epidemiol Infect	2002 – 2009	Hull and East Yorkshire Hospitals[c]	Inpatients only	≥16 y	ICD-10 codes (1st position only): J18.0, J18.9, J13X, J18.1, and J15X	HAP included	Mid-year population estimates for Hull (city) and EroY (Surrounding County) from Office for National Statistics	143 (2002) – 207 (2009)	15 –64	48.8 – 84.1	Fewer ICD-10 codes included than other analyses; Y95 Nosocomial infection included.
									≥65	543 – 781	
Millet 2013, J Clin Epidemiol	1997 – 2011	UK	Both inpatients and outpatients	≥65 y	Read and ICD 10 codes; no specified codes provided. For ICD-10, used first diagnosis code for first episode of hospitalization only.	HAP Excluded	Mid-year UK population estimates from Office for National Statistics	799	65 –69	281	Incidence estimates converted to per 100,000 person-years
									70 –74	431	
									75 –79	694	
									80 –84 y	1,205	
									85 –89	2,184	
									≥90	4,194	
Pick 2020, Thorax[a]	2013 – 2014	Nottingham (2 large university hospitals)	Inpatients only	≥16 y	Inclusion criteria: one or more symptom suggestive of LRTI (defined as cough, increasing dyspnea, sputum production and fever), with evidence of acute infiltrates consistent with respiratory infection on admission radiography, and treated for a diagnosis of CAP Exclusion criteria: hospitalization within 10 days of index admission, a diagnosis of tuberculosis or post-obstructive pneumonia.	HAP Excluded	Mid-year estimates for the Greater Nottingham area from the Office for National Statistics, including local population data stratified by age group	96.3	16 –49	27.3	Only consented/enrolled subjects included in estimates
									50 –64	80.2	
									65 –74	181.3	Required CXR -confirmation but not all LRTI patients had CXR
									75 –84	400.6	
									≥85	707.5	Census-derived denominator that may not have fully matched catchment area.
	2017 – 2018							158.4	16 –49	29.9	
									50 –64	146.9	

For peer review only

For peer review only - http://bmjopen.bmj.com/site/about/guidelines.xhtml

									65 –74	310.4	Required specific symptoms and evidence of treatment and some CAP events may not have had this information documented
									75 –84	559.5	
									≥85	1522.6	
	2013 – 2018							120.4	--	--	
Thorrington 2019, BMC Med	2004 – 2005	England	Inpatients only	≥65 y	ICD 10 codes (1st position only): J18 (pneumonia of unspecified causative organism)	HAP Included	Mid-year population estimates for England for 2004 to 2015 from Office for National Statistics	NA	≥65	829	Incidence is per 100,000 person-years. Fewer ICD-10 codes included than other analyses
	2014 – 2015								≥65	1787	
Trotter 2008, EID	1997 – 1998	England	Inpatients only	≥65 y	ICD 10 codes (1st position only): J12 – J18	HAP Included	Mid-year population estimates for England for 1997 to 2004 from the Office for National Statistics	NA	65 –74	263	Incidence estimates converted to 100,000 population
									75 –84	684	
									≥85	1599	
	2004 – 2005								65 –74	355	
									75 –84	877	
									≥85	2218	
**Lower Respiratory Tract Infection**						**Pneumonia**					
Current Study	2018 – 2019	Bristol (Southmead Hospital)	Inpatients only	≥18 y	Clinical signs/symptoms of heart failure or elevated pro-NT BNP or radiological change **AND** Retrospective ICD-10 code analysis: J09, J10, J11; J20, J21, J22, J40, J41, J42, J44, J45, and J46	Excludes all Pneumonia	Based on number of persons ≥18 years registered in referring GP practices. For practices with split referral patterns, number adjusted for percent of admissions that came to Southmead.	802	18 –49	254	
									50 –64	448	
									65 –74	1,252	
								739	75 –84	2,442	
									≥85	6,116	
Lovering 2001, Clinical Micro. & Infection	1994 – 1996	Bristol (Southmead Hospital)	Inpatients only	≥16 y	LRTI episodes from four groups by ICD 9/10 codes: (1) CAP; (2) chest infection or acute exacerbation in presence of asthma; (3) chest infection or acute exacerbation in presence of COPD; or (4) bronchitis with no radiological evidence of pneumonia or pre-existing respiratory disease, such as COPD or asthma. No specified codes provided.	Includes Community-acquired Pneumonia	No information on denominator provided	623	16 –39	151	Incidence converted to per 100,000 population
									40 –49	175	
									50 –59	294	Study involved single hospital and no mention of source of denominator mentioned.
									60 –69	1,086	
									70 –79	2,135	
						HAP Excluded			>79	3,141	
Millet	1997 –	UK	Both	≥65 y	Read and ICD 10 codes; no specified	Includes	Mid-year UK population	12,293	65 –69	9,221	Incidence

For peer review only - http://bmjopen.bmj.com/site/about/guidelines.xhtml

2013, J Clin Epidemiol	2011		inpatients and outpatients		codes provided. For ICD-10, used first diagnosis code for inpatient episode only.	Community-acquired Pneumonia<br><br>HAP Excluded	estimates from Office for National Statistics. (Patients were not considered at risk for community-acquired LRTI during an LRTI illness-episode, during a HES hospitalization, or for 14 days after any HES hospitalization or CPRD hospital code. This person-time was excluded from denominator.)		70 –74<br>75 –79<br>80 –84<br>85 –89<br>≥90	10,740<br>12,607<br>15,137<br>18,791<br>26,287	converted to per 100,000 person years
**Heart Failure**						**All or First Episode**					
Current Study	2018-2019	Bristol (Southmead Hospital)	Inpatients only	≥18 y	Clinical signs/symptoms of heart failure or elevated pro-NT BNP or radiological change<br><br>**AND**<br><br>Retrospective ICD-10 code analysis: I110; I130; I132; I50	All	Based on number of persons ≥18 years registered in referring GP practices. For practices with split referral patterns, number adjusted for percent of admissions that came to Southmead.	328<br><br>402	18 –49<br>50 –64<br>65 –74<br>75 –84<br>≥85	21<br>206<br>869<br>1,645<br>4,005	
Uijl 2019, Eur J Heart Fail	2000 – 2010	UK	Both inpatients and outpatients	≥55 y	4 sources of HER were linked: CPRD primary care records, HES secondary care hospital charges, Myocardial Ischaemia National Audit Project (MINAP) disease registry, and ONS national death registry. HES ICD-10 codes: Heart failure: I110, I130, I132, I260, I50 and I21. Individuals were excluded if they presented a history of HF before their index date in CPRD, HES or MINAP.	First episode at 55 years or older counted	Not Reported	Not Reported	55 –64, M<br>55 –64, F<br>65 –74, M<br>65 –74, F<br>>75, M<br>>75, F	360<br>190<br>1,360<br>920<br>3,440<br>2,800	Incidence converted to per 100,000 person years<br>Included first episode of HF (inpatient or outpatient) at age 55 and up, so repeat episodes not included.

[a] Only the most recent incidence estimates from the Nottingham CAP study were included.
[b] Add text names for all listed ICD codes in this footnote or appendix.
[c] Included hospitals were Hull Royal Infirmary, Castle Hill Hospital, Princess Royal Hospital, Scarborough District General Hospital, Bridlington & District Hospital, York Teaching Hospital, Scunthorpe General Hospital, Goole & District Hospital.
[d] For the current study, only age-group estimates from the retrospective analysis were included in this table, but these were closely comparable to the annualized incidence estimates based on the prospective review. See Table 3 for full results.

For peer review only - http://bmjopen.bmj.com/site/about/guidelines.xhtml

Figure Legends:

Figure 1: aLRTD admissions identified by Retrospective ICD-10 diagnostic code analysis at North Bristol National Health Service Trust— United Kingdom 2018–2019.

Monthly number of patients admitted, based on ICD-10 coding analysis, with (A) all acute Lower Respiratory Tract Disease (aLRTD) (black bars), (B) Pneumonia (slashed bars), (C) Non-pneumonic Lower Respiratory Tract Infection (NP-LRTI) (white bars), (D) Other LRTD (cross-hash bars), and (E) Heart Failure (grey bars).

Figure 2: Flow diagram of the Prospective Review

For peer review only

For peer review only - http://bmjopen.bmj.com/site/about/guidelines.xhtml

For peer review only

For peer review only - http://bmjopen.bmj.com/site/about/guidelines.xhtml

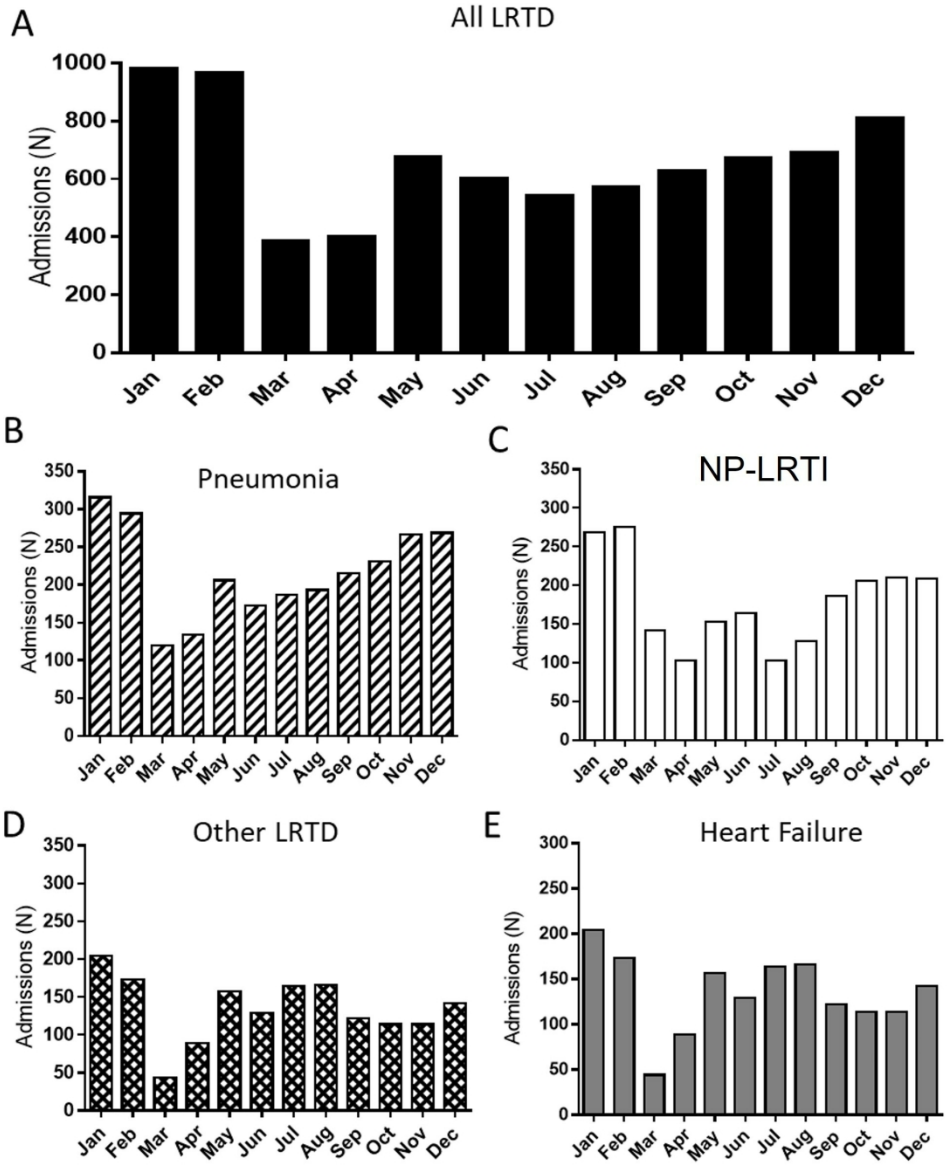

Figure 1: aLRTD admissions identified by Retrospective ICD-10 diagnostic code analysis at North Bristol National Health Service Trust— United Kingdom 2018–2019. Monthly number of patients admitted, based on ICD-10 coding analysis, with (A) all acute Lower Respiratory Tract Disease (aLRTD) (black bars), (B) Pneumonia (slashed bars), (C) Non-pneumonic Lower Respiratory Tract Infection (NP-LRTI) (white bars), (D) Other LRTD (cross-hash bars), and (E) Heart Failure (grey bars).

169x207mm (300 x 300 DPI)

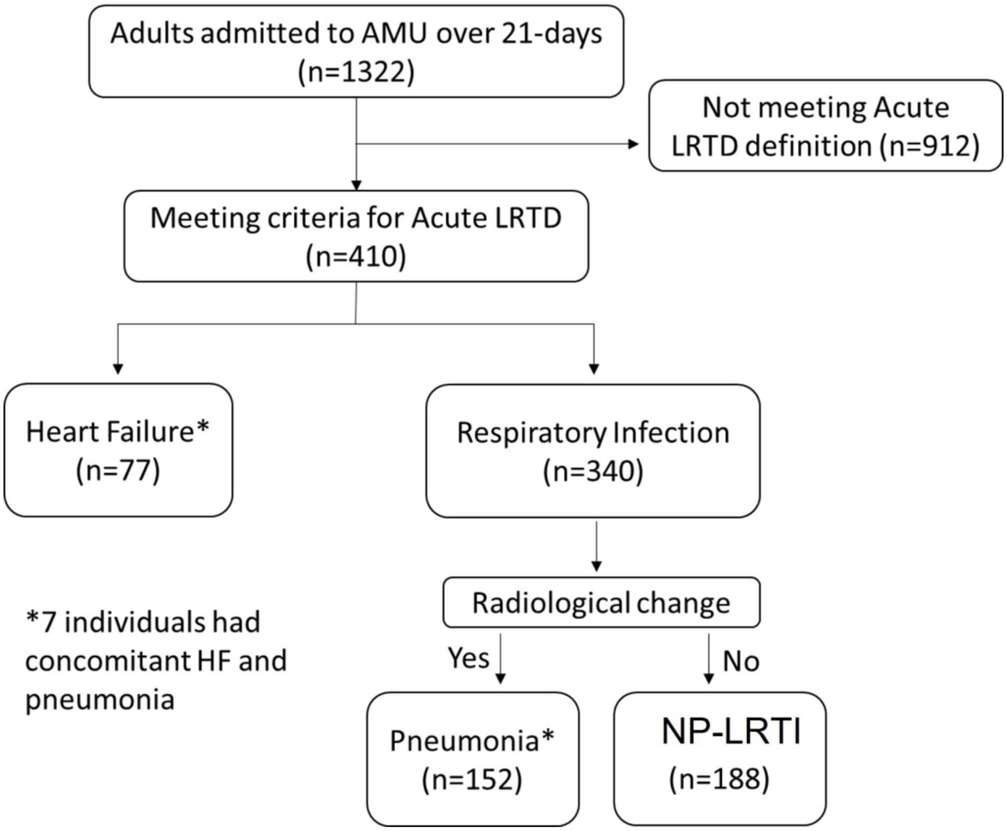

Figure 2: Flow diagram of the Prospective Review

149x123mm (300 x 300 DPI)

Supplementary Data 1: ICD-10 codes used for patient identification for Retrospective Analysis

ICD-10 code	Definition	Pneumonia	NP-LRTI	HF	Other aLRTD
J09	Influenza due to identified zoonotic or pandemic influenza virus		X		
J10	Influenza due to identified seasonal influenza virus		X		
J11	Influenza, virus not identified		X		
J12	Viral pneumonia, not elsewhere classified	X			
J13	Pneumonia due to *Streptococcus pneumoniae*	X			
J14	Pneumonia due to *Haemophilus influenzae*	X			
J15	Bacterial pneumonia, not elsewhere classified	X			
J16	Pneumonia due to other infectious organisms, not elsewhere classified	X			
J17	Pneumonia in diseases classified elsewhere	X			
J18	Pneumonia, organism unspecified	X			
J20	Acute bronchitis		X		
J21	Acute bronchiolitis		X		
J22	Unspecified acute lower respiratory infection		X		
J40	Bronchitis, not specified as acute or chronic		X		
J41	Simple and mucopurulent chronic bronchitis		X		
J42	Unspecified chronic bronchitis		X		
J43	Emphysema				X
J44	Other chronic obstructive pulmonary disease (including J44.0 chronic		J44.0 only		X (J44.0)

	obstructive pulmonary disease with acute lower respiratory infection)				
J45	Asthma				X
J46	Status asthmaticus				X
J47	Bronchiectasis				X
J85	Abscess of lung and mediastinum		X		
J86	Pyothorax		X		
J90	Pleural effusion, not elsewhere classified				X
J91	Pleural effusion in conditions classified elsewhere				X
J95	Postprocedural respiratory disorders, not elsewhere classified				X
J96	Respiratory failure, not elsewhere classified				X
J98	Other respiratory disorders				X
J99	Respiratory disorders in diseases classified elsewhere				X
I110	Hypertensive heart disease with heart failure			X	
I130	Hypertensive heart and chronic kidney disease with heart failure and stage 1 through stage 4 chronic kidney disease			X	
I132	Hypertensive heart and chronic kidney disease with heart failure and with stage 5 chronic kidney disease, or end stage renal disease			X	
I50	Heart failure			X	

For peer review only

Supplementary Data 2: Case Definitions

Condition	Definition	Reference
Acute Lower Respiratory Tract Disease (aLRTD)	Acute lower respiratory tract disease (aLRTD) encompasses pneumonia, non-pneumonic lower respiratory tract infection (LRTI), acute bronchitis, exacerbation of underlying respiratory disease including asthma and chronic obstructive pulmonary disease (COPD), and cardiac failure with respiratory symptoms.<br><br>Pneumothorax, pulmonary embolism, progression or new diagnosis of primary or secondary lung malignancy were excluded from aLRTD.	
Pneumonia	Pneumonia was defined as infection affecting the airways (below the level of the larynx), with either:<br>(1) an acute illness with radiographic shadowing which was at least segmental or present in more than one lobe and was not known to be previously present or due to other causes<br>(2) in the absence of radiological investigation, clinical confirmation of pneumonic disease in the opinion of the treating physician	Lim WS, Baudouin SV, George RC, *et al*; Pneumonia Guidelines Committee of the BTS Standards of Care Committee. BTS guidelines for the management of community acquired pneumonia in adults: update 2009. Thorax. 2009 Oct;64 Suppl 3:iii1-55.
Non-Radiologically Proven Lower Respiratory Tract Infection (NP-LRTI)	An infection that affects the airways (below the level of the larynx) including the trachea and alveoli, with neither the presence of radiological change nor a clinical diagnosis of pneumonia from the treating physician, i.e. non-pneumonic infection in the lungs.	Anderson W, Winter J. Managing LRTI in adults in the community. Practitioner. 2009 Nov;253(1723):21-5, 2-3. PMID: 20043506.
Cardiac/Heart Failure (HF)	A clinical syndrome with symptoms and/or signs caused by a structural and/or functional cardiac abnormality and corroborated by elevated natriuretic peptide levels and or objective evidence of pulmonary or systemic congestion.	Bozkurt, Biykem et al.Universal Definition and Classification of Heart Failure. Journal of Cardiac Failure, Volume 27, Issue 4, 387 – 413.
Other aLRTD	aLRTD which was neither pneumonia, NP-LRTI nor HF was classified as 'Other aLRTD'. This therefore includes non-infective exacerbations of chronic respiratory disease such as asthma, COPD and bronchiectasis	

For peer review only

STROBE Statement—checklist of items that should be included in reports of observational studies

	Item No	Recommendation	Page No
**Title and abstract**	1	(*a*) Indicate the study's design with a commonly used term in the title or the abstract	3
		(*b*) Provide in the abstract an informative and balanced summary of what was done and what was found	3
**Introduction**			
Background/rationale	2	Explain the scientific background and rationale for the investigation being reported	5-6
Objectives	3	State specific objectives, including any prespecified hypotheses	6
**Methods**			
Study design	4	Present key elements of study design early in the paper	7
Setting	5	Describe the setting, locations, and relevant dates, including periods of recruitment, exposure, follow-up, and data collection	7-9
Participants	6	(*a*) *Cohort study*—Give the eligibility criteria, and the sources and methods of selection of participants. Describe methods of follow-up<br>*Case-control study*—Give the eligibility criteria, and the sources and methods of case ascertainment and control selection. Give the rationale for the choice of cases and controls<br>*Cross-sectional study*—Give the eligibility criteria, and the sources and methods of selection of participants	7-9
		(*b*) *Cohort study*—For matched studies, give matching criteria and number of exposed and unexposed<br>*Case-control study*—For matched studies, give matching criteria and the number of controls per case	
Variables	7	Clearly define all outcomes, exposures, predictors, potential confounders, and effect modifiers. Give diagnostic criteria, if applicable	8
Data sources/ measurement	8*	For each variable of interest, give sources of data and details of methods of assessment (measurement). Describe comparability of assessment methods if there is more than one group	7-8
Bias	9	Describe any efforts to address potential sources of bias	15-16
Study size	10	Explain how the study size was arrived at	7-8
Quantitative variables	11	Explain how quantitative variables were handled in the analyses. If applicable, describe which groupings were chosen and why	8-9
Statistical methods	12	(*a*) Describe all statistical methods, including those used to control for confounding	9
		(*b*) Describe any methods used to examine subgroups and interactions	NA
		(*c*) Explain how missing data were addressed	NA
		(*d*) *Cohort study*—If applicable, explain how loss to follow-up was addressed<br>*Case-control study*—If applicable, explain how matching of cases and controls was addressed<br>*Cross-sectional study*—If applicable, describe analytical methods taking account of sampling strategy	NA
		(*e*) Describe any sensitivity analyses	NA

Continued on next page

For peer review only - http://bmjopen.bmj.com/site/about/guidelines.xhtml

**Results**			
Participants	13*	(a) Report numbers of individuals at each stage of study—eg numbers potentially eligible, examined for eligibility, confirmed eligible, included in the study, completing follow-up, and analysed	10, 21-24
		(b) Give reasons for non-participation at each stage	NA
		(c) Consider use of a flow diagram	24
Descriptive data	14*	(a) Give characteristics of study participants (eg demographic, clinical, social) and information on exposures and potential confounders	21-24
		(b) Indicate number of participants with missing data for each variable of interest	NA
		(c) *Cohort study*—Summarise follow-up time (eg, average and total amount)	NA
Outcome data	15*	*Cohort study*—Report numbers of outcome events or summary measures over time	10-11, 21-24
		Case-control study—Report numbers in each exposure category, or summary measures of exposure	*NA*
		Cross-sectional study—Report numbers of outcome events or summary measures	*NA*
Main results	16	(*a*) Give unadjusted estimates and, if applicable, confounder-adjusted estimates and their precision (eg, 95% confidence interval). Make clear which confounders were adjusted for and why they were included	11
		(*b*) Report category boundaries when continuous variables were categorized	NA
		(*c*) If relevant, consider translating estimates of relative risk into absolute risk for a meaningful time period	NA
Other analyses	17	Report other analyses done—eg analyses of subgroups and interactions, and sensitivity analyses	10, 21-25
**Discussion**			
Key results	18	Summarise key results with reference to study objectives	12
Limitations	19	Discuss limitations of the study, taking into account sources of potential bias or imprecision. Discuss both direction and magnitude of any potential bias	15
Interpretation	20	Give a cautious overall interpretation of results considering objectives, limitations, multiplicity of analyses, results from similar studies, and other relevant evidence	12-16
Generalisability	21	Discuss the generalisability (external validity) of the study results	12-16
**Other information**			
Funding	22	Give the source of funding and the role of the funders for the present study and, if applicable, for the original study on which the present article is based	1

*Give information separately for cases and controls in case-control studies and, if applicable, for exposed and unexposed groups in cohort and cross-sectional studies.

Note: An Explanation and Elaboration article discusses each checklist item and gives methodological background and published examples of transparent reporting. The STROBE checklist is best used in conjunction with this article (freely available on the Web sites of PLoS Medicine at http://www.plosmedicine.org/, Annals of Internal Medicine at http://www.annals.org/, and Epidemiology at http://www.epidem.com/). Information on the STROBE Initiative is available at www.strobe-statement.org.

For peer review only - http://bmjopen.bmj.com/site/about/guidelines.xhtml

The incidence of acute lower respiratory tract disease hospitalizations, including pneumonia, among adults in Bristol, UK, 2019, estimated using both a prospective and retrospective methodology

Journal:	*BMJ Open*
Manuscript ID	bmjopen-2021-057464.R2
Article Type:	Original research
Date Submitted by the Author:	27-May-2022
Complete List of Authors:	Hyams, Catherine; University of Bristol, Academic Respiratory Unit; University of Bristol, Bristol Vaccine Centre<br>Begier, Elizabeth; Pfizer Inc<br>Garcia Gonzalez, Maria; University of Bristol; Southmead Hospital, Academic Respiratory Unit<br>Southern, Jo; Pfizer Ltd<br>Campling, James; Pfizer Ltd<br>Gray, Sharon; Pfizer Inc<br>Oliver, Jennifer; University of Bristol, Schools of Cellular and Molecular Medicine and Population Health Sciences<br>Gessner, Bradford; Pfizer Inc<br>Finn, Adam; University of Bristol, Bristol Vaccine Centre
**Primary Subject Heading**:	Respiratory medicine
Secondary Subject Heading:	Infectious diseases, Epidemiology
Keywords:	Heart failure < CARDIOLOGY, Respiratory infections < THORACIC MEDICINE, Epidemiology < INFECTIOUS DISEASES

I, the Submitting Author has the right to grant and does grant on behalf of all authors of the Work (as defined in the below author licence), an exclusive licence and/or a non-exclusive licence for contributions from authors who are: i) UK Crown employees; ii) where BMJ has agreed a CC-BY licence shall apply, and/or iii) in accordance with the terms applicable for US Federal Government officers or employees acting as part of their official duties; on a worldwide, perpetual, irrevocable, royalty-free basis to BMJ Publishing Group Ltd ("BMJ") its licensees and where the relevant Journal is co-owned by BMJ to the co-owners of the Journal, to publish the Work in this journal and any other BMJ products and to exploit all rights, as set out in our licence.

The Submitting Author accepts and understands that any supply made under these terms is made by BMJ to the Submitting Author unless you are acting as an employee on behalf of your employer or a postgraduate student of an affiliated institution which is paying any applicable article publishing charge ("APC") for Open Access articles. Where the Submitting Author wishes to make the Work available on an Open Access basis (and intends to pay the relevant APC), the terms of reuse of such Open Access shall be governed by a Creative Commons licence – details of these licences and which Creative Commons licence will apply to this Work are set out in our licence referred to above.

Other than as permitted in any relevant BMJ Author's Self Archiving Policies, I confirm this Work has not been accepted for publication elsewhere, is not being considered for publication elsewhere and does not duplicate material already published. I confirm all authors consent to publication of this Work and authorise the granting of this licence.

For peer review only

Title: The incidence of acute lower respiratory tract disease hospitalizations, including pneumonia, among adults in Bristol, UK, 2019, estimated using both a prospective and retrospective methodology

Authors: Catherine Hyams[1,2,3], Elizabeth Begier[4], Maria Garcia Gonzalez[1,2], Jo Southern[5], James Campling[5], Sharon Gray[4], Jennifer Oliver[1], Bradford D. Gessner[4], Adam Finn[1]

Affiliations:

[1] Bristol Vaccine Centre, Schools of Cellular and Molecular Medicine and Population Health Sciences, University of Bristol, Bristol, BS2 8AE, United Kingdom

[2] Academic Respiratory Unit, Learning and Research Building, Southmead Hospital, Bristol, UK, BS10 5NB

[3] Acute Medical Unit, Southmead Hospital, Bristol, UK, BS10 5NB

[4] Vaccines Medical Development, Scientific and Clinical Affairs, Pfizer Inc, Collegeville, PA, USA

[5] Vaccines Medical Affairs, Pfizer Ltd, Tadworth, UK, KT20 7NS

Corresponding author: Catherine Hyams, MBBS BSc(Hons) PhD
Population Health Sciences, University of Bristol,
Bristol, BS2 8AE,
United Kingdom
catherine.hyams@nbt.nhs.uk
Catherine.hyams@bristol.ac.uk

Alternate author: Maria Garcia Gonzalez
qa18884@bristol.ac.uk

Running Title: Incidence of acute lower respiratory tract disease in adults

Keywords: Pneumonia, respiratory infection, lower respiratory tract infection, heart failure

Funding: CH was funded by the National Institute for Health Research (NIHR) [NIHR Academic Clinical Fellowship (ACF-2015-25-002]. The views expressed are those of the author(s) and not necessarily those of the NIHR or the

For peer review only - http://bmjopen.bmj.com/site/about/guidelines.xhtml

Department of Health and Social Care. The remainder of the study funding was from Pfizer, Inc (WI255886-1).

Competing Interests: EB, JS, JC, SG, and BG are full time employees of Pfizer Vaccines and hold stock or stock options. CH is Principal Investigator of the Avon CAP study which is an investigator-led University of Bristol study funded by Pfizer and has previously received support from the NIHR in an Academic Clinical Fellowship. AF is a member of the Joint Committee on Vaccination and Immunization (JCVI) and chair of the World Health Organization European Technical Advisory Group of Experts on Immunization (ETAGE) committee. In addition to receiving funding from Pfizer as Chief Investigator of this study, he leads another project investigating transmission of respiratory bacteria in families jointly funded by Pfizer and the Gates Foundation. The other authors have no relevant conflicts of interest to declare.

Word count: **2997/4000**

Summary (38/40 words): Using both retrospective and prospective methodologies, we found a high annual incidence of acute lower respiratory disease (>1700 per 100000; 1.7%) in and around Bristol, UK: pneumonia (~0.6%), lower respiratory tract infection without pneumonia (>0.7%), and heart failure (>0.3%).

Author Contributions: CH, EB, MGG, JS, BDG and AF generated the research questions and analysis plan CH, EB, MGG, JS, JC, SG and BDG undertook and contributed to the data analysis. AF oversaw the research and data collection which was undertaken by CH and MGG. All authors contributed to the preparation of the manuscript.

For peer review only - http://bmjopen.bmj.com/site/about/guidelines.xhtml

For peer review only

Abstract (278/300):

Objectives

To determine the disease burden of acute lower respiratory tract disease (aLRTD) and its subsets (pneumonia, lower respiratory tract infection [LRTI], heart failure) in hospitalized adults in Bristol, UK.

Setting

Single-centre, secondary care hospital, Bristol UK

Design

We estimated aLRTD hospitalizations incidence in adults (≥18 years) in Bristol, UK using two approaches. First, retrospective ICD-10 code analysis (first five positions/hospitalization) identified aLRTD events over a 12-month period (March2018–Feb2019). Secondly, during a 21-day prospective review (19Aug–9Sept2019), aLRTD admissions were identified, categorized by diagnosis, and subsequently annualized. Hospital catchment denominators were calculated using linked general practice and hospitalization data, with each practice's denominator contribution calculated based on practice population and percent of the practices' hospitalizations admitted to the study hospital.

Participants

Prospective review: 1322 adults screened; 410 identified with aLRTD. Retrospective review: 7727 adult admissions.

Primary and Secondary outcome measures

The incidence of aLRTD and its subsets in the adult population of Southmead Hospital, Bristol UK.

Results

Based on ICD-10 code analysis, annual incidences per 100000 population were: aLRTD, 1901; pneumonia, 591; LRTI, 739; heart failure, 402. aLRTD incidence was highest among those ≥65-years: 65–74 (3684 per 100000 adults), 75–84 (6962 per 100000 adults), and ≥85 (11430 per 100000 adults).

For peer review only - http://bmjopen.bmj.com/site/about/guidelines.xhtml

During the prospective review, 410/1322 (31%) hospitalized adults had aLRTD signs/symptoms, and annualized incidences closely replicated retrospective analysis results.

Conclusions

aLRTD disease burden was high, increasing sharply with age. aLRTD incidence is probably higher than estimated previously due to criteria specifying respiratory-specific symptoms or radiological change, usage of only the first diagnosis code, and mismatch between case count sources and population denominators. This may have significant consequences for healthcare planning, including usage of current and future vaccinations against respiratory infection.

Strengths and Limitations of This Study

- We used two analytical methods at the same site over a comparable period, to calculate incidence using both prospective and retrospective approaches.
- The case burden of aLRTD and its subgroups was pre-defined and included patients with atypical presentations
- We calculated incidence using a denominator derived from GP records, providing increased accuracy compared to population calculations based on census data.
- This was a single-center study, with a predominantly Caucasian cohort; therefore, the findings might not be generalizable to other populations.
- The ICD-10 coding data analysis was limited to codes within the first five positions, and therefore may have excluded some cases where other diagnoses were placed higher in the diagnostic coding hierarchy.

For peer review only - http://bmjopen.bmj.com/site/about/guidelines.xhtml

Introduction

Acute lower respiratory tract disease (aLRTD) encompasses pneumonia, non-pneumonic lower respiratory tract infection (NP-LRTI), acute bronchitis, exacerbation of underlying respiratory diseases (including asthma and chronic obstructive pulmonary disease [COPD]), and acute heart failure (HF) events resulting in respiratory symptoms (e.g. breathlessness). Before the COVID-19 pandemic, European healthcare costs for pneumonia alone in adults were estimated at €10 billion annually, including €5.7 billion for inpatient care. [1] Pneumonia incidence in Europe varies by country and intra-country region, age, socioeconomic status, and gender; [2-4] however, in all studies pneumonia incidence in adults increases sharply with age. [3] Pneumonia affects an estimated 0.5% to 1% of UK adults each year [5, 6]. Overall LRTI incidence is considerably higher with ~15% of UK adults aged ≥65 years experiencing an event each year.[7] Whilst HF is not typically clinically included as an acute respiratory illness, HF with respiratory symptoms may be caused by respiratory viral infection, such as respiratory syncytial virus (RSV), either acutely or 3-4 weeks after the primary infection [8, 9].

However, aLRTD incidence may be considerably higher than previously reported, given that published literature has documented several reasons why previous estimates may have been erroneously low [1]. Estimates of aLRTD based on pneumonia defined as radiologically demonstrated alveolar infiltrates, may underestimate true disease burden as chest radiography (CXR) is an imperfect gold-standard. [10, 11] Immunosuppressed, elderly or dehydrated patients are likely to be under-represented if respiratory infection is defined by radiologically demonstrated changes. [10, 11] Microbiological investigations for pneumonia are undertaken variably and identify a causative pathogen in 50% of cases at most [12, 13]; hence, the disease is probably under-reported when confirmed microbiological diagnosis is required. Furthermore, Respiratory Syncytial Virus (RSV) infection has recently been recognized as an important respiratory pathogen later in life, [9] with severe disease occurring in patient groups in whom the diagnosis is likely to be under-recognized (e.g.

For peer review only

For peer review only - http://bmjopen.bmj.com/site/about/guidelines.xhtml

the elderly or those with underlying cardiac conditions). [8] Studies of clinical coding data are retrospective and subject to recognized limitations associated with this methodology [14, 15]. Older patients with pneumonia often have atypical presenting signs and symptoms, which may lead to missed or incorrect admission diagnoses. [16] Pneumonia may occur secondary to, or be an underlying cause of, the main presenting complaint, particularly in patients with cerebrovascular accidents (CVA), HF, COPD exacerbations or altered consciousness levels. [17] In these scenarios, pneumonia may not be the primary hospitalization diagnosis code and may not even be coded as an associated diagnosis.

There are many studies examining the incidence of acute respiratory illness in children; however, data on respiratory illness in adults in the UK is lacking. Given the paucity of data supporting accurate aLRTD incidence rates and its disease subsets in adults, we undertook to assess aLRTD incidence by two approaches (retrospective and prospective) in Bristol, UK, seeking to determine the disease burden of hospitalized aLRTD and its subgroups more accurately.

For peer review only

For peer review only - http://bmjopen.bmj.com/site/about/guidelines.xhtml

Methods

Study Design

This study was conducted at a large secondary care institution in UK (North Bristol NHS Trust) with specialist Respiratory Services (interstitial lung disease, pleural disease). Two approaches were undertaken to estimate aLRTD incidence: (1) “retrospective analysis” of aLRTD International Classification of Diseases Tenth Revision (ICD-10) diagnostic codes for an entire year; and, (2) 21-day observational “prospective review” of aLRTD hospital admissions.

Ethics

This study was approved by North Bristol NHS Trust Research Audit Ethics Committee (CA52218). Data were anonymized before the authors accessed them for the purpose of this study.

Patient and Public Involvement

No patient involved.

Retrospective Analysis

For the retrospective analysis, all adult inpatient admissions (≥18 years) obtained from Hospital Episode Statistic (HES) to the study hospital during March 2018–February 2019 with aLRTD ICD-10 diagnostic codes (Supplementary data 1) in any of the first 5 positions were identified and categorized into aLRTD subgroups: pneumonia, NP-LRTI, other LRTD, and HF (Supplementary data 2). A mutually exclusive hierarchy was used (pneumonia, NP-LRTI, then other LRTD) although HF diagnoses could co-occur with other categories. “Other LRTD” included acute respiratory events that could not definitively be placed in another category. Only the first 5 ICD-10 codes were available for analysis.

Prospective Review

Adult patients (≥18 years) resident within Bristol, North Somerset, and South Gloucestershire Clinical Commissioning Group (CCG) referred to the Acute Medical Unit (AMU) at North Bristol NHS Trust

For peer review only - http://bmjopen.bmj.com/site/about/guidelines.xhtml

during 19th August –9th September 2019 were included in an audit on acute respiratory illness. This time period was selected because it was felt to represent a period when there were an average number of adults hospitalised with aLRTD. A respiratory physician (CH) reviewed presenting features and investigation results for each admitted patient to determine whether aLRTD was present. Further medical record review was undertaken if patients had: new/worsening breathlessness, cough or sputum production; new or worsening peripheral oedema; pleurisy; clinical examination findings consistent with respiratory infection or HF; or fever attributable to suspected respiratory infection. Patients with non-respiratory diagnoses were excluded. There were no patient refusals for either approach.

Prospective Review Outcome measures

aLRTD was considered confirmed in individuals with: new/worsening respiratory symptoms, with or without fever; sepsis, delirium or raised inflammatory markers attributable to likely respiratory infection in admitting clinical team's opinion; radiological change in keeping with infection (e.g. consolidation); and/or final diagnosis of NP-LRTI, pneumonia or infective exacerbation of a chronic respiratory condition. A pneumonia diagnosis was assigned if radiological changes likely due to infection were described by the reporting radiologist. An NP-LRTI diagnosis was assigned if aLRTD signs and symptoms likely to be due to infection were present without demonstrated radiological change. A HF diagnosis was assigned in presence of: new/worsening breathlessness and bi-basal crepitations, cardiac wheeze or new/worsening bilateral pitting oedema; elevated pro-NT BNP (≥450pg/mL); radiologist-reported radiographic changes consistent with cardiac failure; or a final consultant physician diagnosis of HF, cardiac failure or left-ventricular failure. When present, ≥1 diagnosis was selected.

For both retrospective and prospective studies, pneumonia included both community and healthcare setting acquired cases; although, the prospective review only captured admitting diagnoses and pneumonias occurring later during hospitalization were not included.

For peer review only

For peer review only - http://bmjopen.bmj.com/site/about/guidelines.xhtml

Incidence calculations

Annual incidence per 100000 persons was calculated for both retrospective and prospective studies. Case counts from prospective review were annualized (i.e., case counts by diagnosis and overall were divided by the percentage of annual admissions for these diagnosis groups that occurred in this 21-day period in the retrospective analysis).

Incidence Denominators

To calculate appropriate population denominators for incidence calculations, aLRTD hospital admission event data were linked to aggregated GP practice patient registration data within the NHS Bristol, North Somerset, and South Gloucestershire CCG for 2017–2019. Only 4% of patients sought care at North Bristol NHS Trust hospital from outside these local CCGs, despite presence of specialist respiratory services. In the UK, GP registration is available free of charge for all, regardless of residential status. For GP practices within these same CCGs, the proportion of their aLRTD admissions occurring at North Bristol NHS Trust was multiplied by their patient registration count in 2019 by age group, to get each practice's contribution to the denominator (e.g. if 50% aLRTD admissions were at North Bristol among persons 50−64 years, the practice would contribute half of their patients 50–64 years to the denominator). Further details of this methodology have been described previously. [18]

Statistical analysis

Patient characteristics were tabulated by aLRTD diagnosis. Categorical variables were presented as counts with percentages. Continuous data are presented with means and standard deviations (SD) if normally distributed and medians and interquartile range (IQR) if not normally distributed. Patient groups difference were evaluated using the Friedman test with Wilcoxon signed-rank test.

For peer review only - http://bmjopen.bmj.com/site/about/guidelines.xhtml

Results

Retrospective analysis

Over a 12-month period, we identified 7727 hospital admissions for aLRTD: 3005 NP-LRTI admissions, 2402 pneumonia, 1633 HF and 1071 other LRTD (Table 1). aLRTD admissions were lowest in March and April and highest December through February (Figure 1A), overall and for all aLRTD subgroups (P<0.05) (Figure 1B-D). Overall, 28.1% (2244) cases were identified as being potentially hospital-acquired infection based on co-occurring ICD-10 code 'Y95 Nosocomial Infection'.

Prospective review

Among 1322 eligible adult patients referred to AMU over the 21-day review period (Figure 2), 410 patients had signs or symptoms of aLRTD: 188 (46%) NP-LRTI; 152 (37%) pneumonia, and 77 (19%) HF. Seven patients had both decompensated HF and a respiratory infection at hospital admission. On admission, >10% of aLRTD patients did not have respiratory symptoms: 16 (11%) pneumonia, 25 (13%) NP-LRTI, and 18 (14%) HF (Table 2).

Almost all adults admitted with aLRTD underwent routine biochemistry, hematology, and radiological investigation (99.9%, n=409). In contrast, only 150 (37%) patients with aLRTD had microbiological testing performed: blood cultures (n=149, 36%) and urine cultures (n=143, 35%). Pneumonia patients more commonly underwent microbiological investigation than NP-LRTI patients ($P<0.05$) with highest disparity in rates of sputum culture, urinary antigens, and respiratory viral PCR (Table 2). All cardiac failure patients who underwent microbiological investigation had concomitant respiratory infection (Table 2). Overall, a microbiological diagnosis was found in 11 (3%) cases, highlighting the low frequency of definitive pathogen identification in aLRTD. Younger patients underwent microbiological testing more frequently than the elderly for all aLRTD categories (Table 2).

For peer review only - http://bmjopen.bmj.com/site/about/guidelines.xhtml

181 *Disease incidence*

182 Retrospective analysis yielded an overall aLRTD incidence of 1901 per 100000. Disease incidence rose
183 with increasing age (Table 3), both overall and for all disease subgroups; incidences per 100000 among
184 adults aged ≥85 years were: 11430 (aLRTD), 6116 (NP-LRTI), 4215 (pneumonia) and 4005 (HF). Overall,
185 28.1% aLRTD hospitalizations also included an ICD-10 discharge code for 'nosocomial infection',
186 suggesting the aLRTD event or other infection was hospital-acquired. For pneumonia 25.3% had the
187 nosocomial infection code. If all of these were related to the pneumonia diagnosis, an estimated
188 residual 1794 events would have been community-acquired pneumonia (annual incidence
189 441/100000 adults). Among older age categories, where the incidence of aLRTD was highest, NP-LRTI
190 incidence was similar to pneumonia incidence, with an approximate 1:1 ratio of NP-LRTI to pneumonia
191 cases. However, among adults under age 50 years, there were approximately twice as many NP-LRTI
192 cases observed as pneumonia cases. Incidence calculations using annualized prospective review
193 results were broadly comparable with retrospective analysis of ICD-10 data (Table 3).

For peer review only

For peer review only - http://bmjopen.bmj.com/site/about/guidelines.xhtml

Discussion:

This is the first UK study to assess aLRTD incidence comprehensively. We conducted an analysis of 12-months of hospital admissions by ICD-10 diagnosis code data and a 21-day prospective review at a large academic hospital in southwest England. With both approaches, we found a high annual incidence of aLRTD (>1700 per 100000; 1.7%), pneumonia (~0.6%), NP-LRTI without pneumonia (>0.7%), and HF (>0.4%). Incidences increased sharply in a non-linear manner as age increased above 65 years for all aLRTD categories. These results suggest rates are probably significantly higher than previous disease estimates from the UK (Table 4) but comparable with many results globally, [19, 20] with important consequences for healthcare resources. For example, a recent review highlighted that pneumonia incidences ranged from 1000 to 2500 per 100000 (1–2.5%) among persons aged 65–74 years in Spain, Germany, France, Japan and the US, which are comparable to the >1250 per 100000 (1.3%) reported here. Some of the potential sources of underestimation for other UK incidence studies (Table 4) include: case source and denominator mismatch; use of first position in ICD-code analysis only; inclusion of enrolled subjects in consent-based prospective studies only; requiring specific symptoms and chest x-ray confirmation in order to be classified as pneumonia/aLRTD; and, lastly, the rising incidence of aLRTD.

Comparison with published literature

No studies have reported aLRTD incidence comprehensively in UK hospitalized patients within the last 20 years. However, eight publications report incidence of ≥1 aLRTD subgroup. Seven publications reported community-acquired pneumonia (CAP) incidence (three from Nottingham, UK). For pneumonia, our incidence estimates were three to four-fold higher than other UK inpatient incidence estimates (Table 4) but comparable to estimates from other countries. [19, 20] Only two UK studies from approximately 20 years ago reported NP-LRTI incidence (one with both CAP and NP-LRTIs; Table 4), and only one provided an inpatient estimate. [21] NP-LRTI incidence was approximately 2-fold

For peer review only - http://bmjopen.bmj.com/site/about/guidelines.xhtml

lower than that calculated here, taking into account inclusion of CAP and other NP-LRTI in their estimates. [21, 22] The one UK study reporting HF incidence had methodological differences (i.e., inclusion of outpatients and limiting to initial HF diagnosis) and estimates could not be compared. [23] Close examination of the existing literature methods yielded multiple sources for potential underestimation.

First, for incidence studies that were not countrywide, identifying an appropriate denominator is challenging. Like many other inpatient settings worldwide, UK hospitals' catchment areas for acute treatment are principally driven by geography, but the proportion of any area's residents expected to use the hospital becomes less clear as distance from the hospital increases because catchment areas and populations of different hospitals may overlap. Defining hospital catchment populations based solely on census data cannot account for this variability. Including all geographic areas using the hospital to any extent results in population denominator overestimation and underestimated incidence. Here, we addressed this by calculating population denominators based on hospital utilization behavior from referring General Practices.

Second, other studies used fewer codes in their ICD code analysis: all limited their analyses to events where the diagnostic code was in the first position (Table 4; case definition column), potentially excluding admissions in which pneumonia/NP-LRTI complicated other underlying respiratory diseases, including COPD and asthma. Limiting to first position has been shown to reduce sensitivity for pneumonia events by about 30% (66%–72% sensitive). [22, 24] Conversely, the recent British Thoracic Society (BTS) audit on CAP found ~27% of pneumonia events identified by ICD code (J12-18) had no new CXR infiltrates. [6] Even accounting for this potential over coding practice, our estimates remain well above other published UK estimates.

Third, for other prospective studies, exclusion of events where patients did not consent to participation or were not identified by study surveillance processes (often conducted predominately during business hours) can introduce underestimation. Further, other prospective pneumonia studies

For peer review only

For peer review only - http://bmjopen.bmj.com/site/about/guidelines.xhtml

specifically required documentation of specific symptoms, radiological findings, and treatments, [25] potentially excluding those without these features documented in medical records. In our prospective review, approximately 11% did not display typical signs and symptoms of pneumonia and could have been excluded by that requirement. Requiring CXR confirmation has been shown to reduce incidence estimates for pneumonia, [20] although all pneumonia events in our prospective review were radiologically confirmed.

Fourth, trends over time may also contribute to our estimates being higher than previous reports. Our study's estimates are recent, and rising incidence of pneumonia has been documented in all studies that have reported such trends. [25-27]

Finally, this study included, in part, hospital-acquired pneumonias (HAP), which were excluded from estimates calculated in some other studies (Table 4). The retrospective analysis may have included more nosocomial infection than the prospective review, as the latter was focused on evaluation of patients at admission for aLRTD and would not have reliably captured events that developed during hospitalization. 25.3% pneumonia events included a nosocomial infection code, but this code could relate to any nosocomial infection during that hospitalization. If all these cases were assumed to be hospital-acquired pneumonia, our estimates community-acquired pneumonia incidence would still be well above prior UK estimates: 441/100000 (≥18 years).

While not impacting all-cause aLRTD incidence estimates discussed above, during the prospective review, we found low rates of microbial investigation which prevented us from generating pathogen-specific incidence estimates. Only 52% of patients with radiologically-confirmed pneumonia underwent microbiological testing during hospitalization, with even lower rates in other aLRTD subgroups (41% NP-LRTI and 14% HF). Microbiological testing occurred less frequently as age increased, particularly in NP-LRTI patients. It is possible that, because aLRTD hospitalizations are substantially more common among older persons, less etiologic investigation is performed. Furthermore, clinicians may elect to treat elderly patients with a more pragmatic and less invasive

For peer review only

For peer review only - http://bmjopen.bmj.com/site/about/guidelines.xhtml

approach. Management guidelines do not require specific pathogen identification to inform treatment choice. Presence only of atypical features on presentation (in this series, 13% NP-LRTI and 11% pneumonia cases) may also reduce the likelihood of timely microbiological testing. Low rates of microbiological testing, and consequently of confirmed microbiological diagnosis, may represent a source of underestimation of pathogen-specific disease incidence in patient groups (i.e., testing bias), particularly in elderly patient groups.

Strengths and Limitations of This Study

This study has many strengths. First, this study used two analytical methods at the same site over a comparable period, to calculate incidence using both prospective and retrospective approaches. Second, the case burden of aLRTD and its subgroups was pre-defined and included patients with atypical presentations but with clinical and/or radiological diagnoses, who may otherwise have been excluded from analysis. Additionally, we calculated incidence using a denominator derived from GP records, providing increased accuracy compared to population calculations based on census data.

However, the study also had some limitations. This was a single-center study, with a predominantly Caucasian cohort; therefore, the findings might not be generalizable to other populations both within the UK and in other countries. Different healthcare systems may affect patient treatment preference, and as the NHS provides care which is free at the point of access, the hospitalization rates seen in this study may be different in fee or insurance based healthcare system. Similarly, physician treatment preferences may affect hospitalization rates, and we have not explored these in this analysis. The ICD-10 coding data analysis was limited to codes within the first five positions, and therefore may have excluded some cases where other diagnoses were placed higher in the diagnostic coding hierarchy. Furthermore, we could not determine how many cases of the 28.1% ICD-10 cases also coded with nosocomial infection had hospital-acquired respiratory infection rather than other nosocomial infections.

For peer review only - http://bmjopen.bmj.com/site/about/guidelines.xhtml

For peer review only

Although the denominator used to calculate incidence was derived from GP records, this was still an estimate as there is no precisely defined denominator hospital catchment. We were unable to exclude patients from outside the local CCGs in the retrospective analysis, due to the way ICD-10 data was obtained. However, these patients were excluded from the prospective review and the incidence calculated was comparable, suggesting any effect that patients attending North Bristol NHS Trust from outside the local CCGs have on incidence estimates is minimal. This may be because any effect of travelling or health-seeking behavior is bi-directional: whilst some patients admitted to Southmead hospital were from outside the local area, it is also true that patients with aLRTD within the relevant CCGs would have been admitted to other hospitals. We also acknowledge the 21-day prospective review period was relatively short, not repeated and may not be fully representative of clinical practice and cases throughout the year. This study was conducted before the emergence of COVID-19, and we think these data will be useful in one of two ways in the context of COVID-19: (1) either COVID-19 will become endemic, and the data will reflect the first year before a new normal, or (2) COVID-19 will abate and it will provide an anchor for understanding incidence during a respiratory viral pandemic'

In conclusion, we found similarly high estimates of LRTD incidence using two different approaches, and these estimates were higher than those obtained previously in the UK. Determining if there is a real increase in incidence, or if this estimate is larger due to more accurate methodology including a more accurate denominator will require ongoing comprehensive surveillance. Nonetheless, combining all types of LRTD highlights the high burden for this important and potentially life-threatening disease group. Incidence assessments require close assessments of potential areas of under ascertainment, including unidentified or unenrolled cases in prospective studies, reduced positions or number of ICD-10 codes included for retrospective studies, and population denominator mismatch for all study types. Our prospective review findings highlight the need to consider atypical clinical presentations for pneumonia and the lack of routine microbiological investigation in many

For peer review only - http://bmjopen.bmj.com/site/about/guidelines.xhtml

aLRTD patients required for pathogen-specific aLRTD incidence calculation. Future research should include a fully prospective assessment of aLRTD incidence with comprehensive diagnostic testing across multiple respiratory seasons, to ensure accurate capture of all aLRTD events, particularly in the elderly. Such research should be undertaken given the high and rising aLRTD burden to enable appropriate healthcare planning and identification of interventions which may reduce disease burden.

For peer review only

For peer review only - http://bmjopen.bmj.com/site/about/guidelines.xhtml

325 **Acknowledgements**

326 We would like to acknowledge the assistance of Qi Yan, PhD (Pfizer, Inc.) who provided
327 indispensable medical writing and literature review support for this manuscript and Harvey Walsh
328 Health who performed the hospital denominator calculation used here. For the denominator
329 analysis, Hospital Episode Statistics (HES) Data were re-used with the permission of NHS Digital via
330 Harvey Walsh Limited.

331

332 **Data Sharing**

333 No additional data available

For peer review only

For peer review only - http://bmjopen.bmj.com/site/about/guidelines.xhtml

References

1. Gibson, G.J., et al., *Respiratory health and disease in Europe: the new European Lung White Book.* Eur Respir J, 2013. **42**(3): p. 559-63.
2. Cilloniz, C., et al., *Impact of age and comorbidity on cause and outcome in community-acquired pneumonia.* Chest, 2013. **144**(3): p. 999-1007.
3. Torres, A., et al., *Burden of pneumococcal community-acquired pneumonia in adults across Europe: A literature review.* Respir Med, 2018. **137**: p. 6-13.
4. *European Lung White Book. Pneumonia.* April 25, 2021]; Available from: https://www.erswhitebook.org/chapters/acute-lower-respiratory-infections/pneumonia/#:~:text=pneumonia%20(VAP).-,Incidence,000%20cases%20are%20expected%20annually.
5. *British Lung Foundation. Pneumonia Statistics.* April 25, 2021]; Available from: https://statistics.blf.org.uk/pneumonia.
6. Lim, W. and H. Lawrence, *British Thoracic Society. National Audit Report: Adult Community Acquired Pneumonia Audit 2018-2019. National Audit Period: 1 December 2018 – 31 January 2019*. 2019.
7. Millett, E.R.C., et al., *Incidence of community-acquired lower respiratory tract infections and pneumonia among older adults in the United Kingdom: a population-based study.* PloS one, 2013. **8**(9): p. e75131-e75131.
8. Branche, A.R. and A.R. Falsey, *Respiratory Syncytial Virus Infection in Older Adults: An Under-Recognized Problem.* Drugs & Aging, 2015. **32**(4): p. 261-269.
9. Falsey, A.R. and E.E. Walsh, *Respiratory syncytial virus infection in elderly adults.* Drugs & aging, 2005. **22**(7): p. 577-587.
10. Maughan, B.C., et al., *False-negative chest radiographs in emergency department diagnosis of pneumonia.* R I Med J (2013), 2014. **97**(8): p. 20-3.
11. Self, W.H., et al., *High discordance of chest x-ray and computed tomography for detection of pulmonary opacities in ED patients: implications for diagnosing pneumonia.* Am J Emerg Med, 2013. **31**(2): p. 401-5.
12. Cilloniz, C., et al., *Microbial aetiology of community-acquired pneumonia and its relation to severity.* Thorax, 2011. **66**(4): p. 340-6.
13. Jain, S., et al., *Community-Acquired Pneumonia Requiring Hospitalization.* N Engl J Med, 2015. **373**(24): p. 2382.
14. Mainor, A.J., et al., *ICD-10 Coding Will Challenge Researchers: Caution and Collaboration may Reduce Measurement Error and Improve Comparability Over Time.* Med Care, 2019. **57**(7): p. e42-e46.
15. Burns, E.M., et al., *Systematic review of discharge coding accuracy.* J Public Health (Oxf), 2012. **34**(1): p. 138-48.
16. Henig, O. and K.S. Kaye, *Bacterial Pneumonia in Older Adults.* Infect Dis Clin North Am, 2017. **31**(4): p. 689-713.
17. Fine, M.J., et al., *Prognosis and outcomes of patients with community-acquired pneumonia. A meta-analysis.* JAMA, 1996. **275**(2): p. 134-41.
18. Campling, J., et al., *A novel approach to calculate disease incidence for hospital-based health events in England.* The Lancet, 2021. **398**: p. S30.
19. Chalmers, J., et al., *Community-acquired pneumonia in the United Kingdom: a call to action.* Pneumonia (Nathan), 2017. **9**: p. 15.
20. Shi, T., et al., *Global and Regional Burden of Hospital Admissions for Pneumonia in Older Adults: A Systematic Review and Meta-Analysis.* J Infect Dis, 2020. **222**(Suppl 7): p. S570-S576.
21. Lovering, A.M., et al., *Epidemiology and resource utilization for patients hospitalized for lower respiratory tract infection.* Clin Microbiol Infect, 2001. **7**(12): p. 666-70.

For peer review only

For peer review only - http://bmjopen.bmj.com/site/about/guidelines.xhtml

22. Yu, O., et al., *Classification algorithms to improve the accuracy of identifying patients hospitalized with community-acquired pneumonia using administrative data.* Epidemiology and Infection, 2011. **139**(9): p. 1296-1306.

23. Uijl, A., et al., *Risk factors for incident heart failure in age- and sex-specific strata: a population-based cohort using linked electronic health records.* Eur J Heart Fail, 2019. **21**(10): p. 1197-1206.

24. van de Garde, E.M.W., et al., *International classification of diseases codes showed modest sensitivity for detecting community-acquired pneumonia.* Journal of Clinical Epidemiology, 2007. **60**(8): p. 834-838.

25. Pick, H., et al., *Pneumococcal serotype trends, surveillance and risk factors in UK adult pneumonia, 2013–18.* Thorax, 2020. **75**(1): p. 38.

26. Elston, J.W.T., et al., *Increasing incidence of invasive pneumococcal disease and pneumonia despite improved vaccination uptake: surveillance in Hull and East Yorkshire, UK, 2002–2009.* Epidemiology and Infection, 2012. **140**(7): p. 1252-1266.

27. Thorrington, D., et al., *Elucidating the impact of the pneumococcal conjugate vaccine programme on pneumonia, sepsis and otitis media hospital admissions in England using a composite control.* BMC Medicine, 2018. **16**(1): p. 13.

For peer review only

Table 1: Demographic Characteristics of Patients admitted with aLRTD for 1 Year ICD-10 code Retrospective Analysis and 21-day Prospective Review Period -- 2018–2019

**Characteristic**	**Pneumonia**		**NP-LRTI**		**Heart Failure**		**Other LRTD**	**All LRTD**	
Study	Prospective review	Retrospective analysis	Prospective review	Retrospective analysis	Prospective review	Retrospective analysis	Retrospective Review Only	Prospective review	Retrospective analysis
N	152	2402	188	3005	77	1633	1071	410	7727
Gender, females	61 (40)	1078 (45)	99 (53)	1482 (49)	39 (51)	731 (45)	489 (46)	194 (47)	3780 (49)
**Age**									
Median (IQR), y	80 (67-86)	81 (66-88)	70 (46-87)	69 (45-87)	87 (72-90)	87 (70-90)	74 (53-82)	80 (64-88)	81 (65-90)
18-24	4 (3)	72 (3)	9 (5)	151 (5)	0 (0)	0 (0)	26 (2)	13 (3)	249 (3)
25-34	2 (2)	48 (2)	12 (6)	183 (6)	0 (0)	3 (0)	33 (3)	14 (3)	267 (3)
35-44	6 (4)	97 (4)	14 (7)	209 (7)	2 (3)	10 (1)	59 (6)	22 (5)	375 (5)
45-54	8 (5)	118 (5)	11 (6)	183 (6)	0 (0)	22 (1)	112 (10)	19 (5)	435 (6)
55-64	18 (12)	293 (12)	19 (10)	305 (10)	8 (10)	158 (10)	210 (20)	45 (11)	966 (13)
65-74	34 (22)	501 (21)	32 (17)	549 (18)	10 (15)	199 (12)	223 (21)	75 (18)	1472 (19)
75-84	44 (28)	667 (28)	40 (21)	621 (21)	20 (30)	498 (31)	205 (19)	100 (24)	1991 (26)
≥85	38 (26)	606 (25)	51 (27)	704 (23)	37 (55)	742 (45)	203 (19)	123 (30)	2255 (29)

For peer review only

For peer review only - http://bmjopen.bmj.com/site/about/guidelines.xhtml

Table 2. Clinical Characteristics and Investigations of Patients admitted with aLRTD over 21-day Prospective Review Period in August-September 2020

Characteristic	Pneumonia n=152 (%)	NP-LRTI n=188 (%)	Heart Failure n=77 (%)	All LRTD n=410 (%)
GP	56 (37)	72 (39)	30 (39)	158 (39)
A&E department	93 (61)	100 (54)	45 (58)	238 (58)
Transfer from another unit	2 (1)	13 (7)	0 (0)	15 (4)
Other	1 (1)	1 (1)	2 (3)	4 (1)
**Referral Source**				
Typical features†	136 (89)	163 (87)	63 (82)	355 (87)
Atypical features	16 (11)	25 (13)	14 (18)	55 (13)
- collapse/falls	11 (7)	12 (6)	0 (0)	23 (6)
- confusion	0 (0)	7 (4)	4 (5)	10 (2)
- drowsiness	1 (1)	1 (1)	2 (3)	4 (1)
- off legs/generally unwell	5 (3)	5 (3)	8 (10)	18 (4)
**LRTD Signs and symptoms on referral to AMU**				
Biochemistry	152 (100)	185 (99)	77 (100)	419 (100)
Haematology	152 (100)	185 (99)	77 (100)	419 (100)
Radiology	152 (100)	185 (99)	77 (100)	419 (100)
**Investigations Performed**				
Testing by Age Group				
All patients	79/152 (52)*	77/188 (41)	11/77 (14)	167 (41)
18-24	3/4 (75)	6/9 (66)	0/0 (0)	9/13 (69)
25-34	0/0 (0)	6/12 (50)	0/0 (0)	6/12 (50)
35-44	5/6 (83)	10/14 (71)	2/2 (100)	17/22 (77)
45-54	6/8 (75)	6/11 (55)	0/0 (0)	13/19 (68)
55-64	11/18 (61)	12/19 (63)	5/8 (63)	31/45 (69)
65-74	15/34 (44)	12/32 (38)	1/10 (10)	28/75 (37)
75-84	21/43 (49)	10/40 (25)	2/20 (10)	33/100 (33)
≥85	18/39 (46)	15/51 (19)	1/37 (3)	34/124 (27)
Test performed				
Blood culture	79 (52)	70 (37)	5 (6)	150 (37)
Urine culture	66 (43)	77 (41)	11 (14)	150 (37)
Sputum culture	27 (18)*	7 (4)	2 (3)	35 (9)
BinaxNOW® Pn UAT ‡	29 (19)*	6 (3)	0 (0)	35 (9)
Respiratory virus PCR	16 (11)*	11 (6)	1 (1)	28 (7)
Pleural fluid culture	3 (2)	0 (0)	1 (1)	4 (1)

A&E, Accident and Emergency department; GP, general practitioner; LRTD, lower respiratory tract disease; NP-LRTI, non-pneumonic lower respiratory tract infection; IQR, interquartile range; Pn UAT, pneumococcal urinary antigen test; PCR, polymerase chain reaction.

* $P<0.05$.

† Typical symptoms included cough, breathlessness, increased or discolored sputum production, wheeze, pleurisy, peripheral oedema, hemoptysis, reduced exercise tolerance and/or fever.

‡ BinaxNOW Pn UAT was only performed in accordance with NICE/BTS guidelines.

For peer review only - http://bmjopen.bmj.com/site/about/guidelines.xhtml

Table 3: Incidence of aLRTD resulting in hospital admission based on prospective and retrospective approaches by age group and condition, North Bristol National Health Service Trust— United Kingdom 2018–2019.

	Age Groups					
	**All Adults**	**18-49 y**	**50-64 y**	**65-74 y**	**75-84 y**	**≥85 y**
**Population in 2018**	406481	226920	91534	45705	29487	12835
**Retrospective Analysis of a Year's ICD-10 codes**						
**Annual cases – N (row %)**						
All aLRTD	7,727	1,130 (14)	1,103 (14)	1,684 (22)	2,053 (27)	1,757 (23)
Pneumonia	2,402	264 (11)	288 (12)	589 (25)	720 (30)	541 (22)
NP-LRTI	3,005	576 (19)	410 (14)	572 (19)	662 (22)	785 (26)
Other LRTD	1,071	246 (23)	268 (25)	226 (21)	200 (19)	131 (12)
Heart Failure	1,633	48 (3)	189 (12)	397 (24)	485 (30)	514 (31)
**LRTI/Pneumonia Ratio**	1.3	2.2	1.4	1.0	0.9	1.5
**Incidence (per 100,000)**						
All aLRTD	1,901	497	1,205	3,684	6,962	13,689
Pneumonia	591	116	315	1,289	2,442	4,215
NP-LRTI	739	254	448	1,252	2,245	6,116
Other LRTD	263	108	293	494	678	1,021
Heart Failure	402	21	206	869	1,645	4,005
**21-day Prospective Review (annualized)**						
**Annualized cases – N (row %)**						
All aLRTD	7,885	1,038	962	1,692	2,231	1,962
Pneumonia	2,621	224	397	776	690	534
NP-LRTI	3,857	796	531	653	1,061	816
Heart Failure	2,000	51	205	308	641	795
**LRTI/Pneumonia Ratio**	1.5	3.6	1.3	0.8	1.5	1.5
**Incidence (per 100,000)**						
All aLRTD	1,940	458	1,050	3,703	7,565	15,283
Pneumonia	645	99	433	1,698	2,339	4,164
NP-LRTI	944	351	580	1,429	3,599	6,360
Heart Failure	492	23	224	673	2,174	6,193

LRTI = lower respiratory tract infection; LRTD = lower respiratory tract disease.
Pneumonia category includes community and healthcare acquired. For retrospective ICD-10 based cohort, the following mutually exclusive hierarchy was used to define pneumonia, LRTI, and other LRTD; heart failure event could overlap with other categories. "Other LRTD" contains LRTD events that could not definitively be placed in one of the other respiratory disease categories.

For peer review only - http://bmjopen.bmj.com/site/about/guidelines.xhtml

Table 4: Literature review of aLRTD incidence in hospitalized adults, United Kingdom

Study	Study Years	Location (Facility)	Event Setting	Age	Case Definition[b]	Key inclusion	Denominator Source	Overall Incidence	Age Breakdown (years)	Incidence per 100,000 by age[d]	Comments
**Community –acquired Pneumonia**											
Current Study	2018 – 2019	Bristol (Southmead Hospital)	Inpatients only	≥18 y	Clinical signs/symptoms with radiological change in keeping with infection (prospective review portion) **AND** Retrospective ICD-10 code analysis (1st 5 positions): J12-J18, J85, and J86	Hospital-acquired pneumonia (HAP) included	Based on number of persons ≥18 years registered in referring GP practices. For practices with split referral patterns, number adjusted for percent of admissions that came to Southmead.	648	18 –49	116	Retrospective analysis includes 1st 5 positions
									50 –64	315	
									65 –74	1,289	
								591	75 –84	2,442	
									≥85	4,215	
Elston 2012, Epidemiol Infect	2002 – 2009	Hull and East Yorkshire Hospitals[c]	Inpatients only	≥16 y	ICD-10 codes (1st position only): J18.0, J18.9, J13X, J18.1, and J15X	HAP included	Mid-year population estimates for Hull (city) and EroY (Surrounding County) from Office for National Statistics	143 (2002) – 207 (2009)	15 –64	48.8 – 84.1	Fewer ICD-10 codes included than other analyses; Y95 Nosocomial infection included.
									≥65	543 – 781	
Millet 2013, J Clin Epidemiol	1997 – 2011	UK	Both inpatients and outpatients	≥65 y	Read and ICD 10 codes; no specified codes provided. For ICD-10, used first diagnosis code for first episode of hospitalization only.	HAP Excluded	Mid-year UK population estimates from Office for National Statistics	799	65 –69	281	Incidence estimates converted to per 100,000 person-years
									70 –74	431	
									75 –79	694	
									80 –84 y	1,205	
									85 –89	2,184	
									≥90	4,194	
Pick 2020, Thorax[a]	2013 – 2014	Nottingham (2 large university hospitals)	Inpatients only	≥16 y	Inclusion criteria: one or more symptom suggestive of LRTI (defined as cough, increasing dyspnea, sputum production and fever), with evidence of acute infiltrates consistent with respiratory infection on admission radiography, and treated for a diagnosis of CAP Exclusion criteria: hospitalization within 10 days of index admission, a diagnosis of tuberculosis or post-obstructive pneumonia.	HAP Excluded	Mid-year estimates for the Greater Nottingham area from the Office for National Statistics, including local population data stratified by age group	96.3	16 –49	27.3	Only consented/enrolled subjects included in estimates
									50 –64	80.2	
									65 –74	181.3	Required CXR - confirmation but not all LRTI patients had CXR
									75 –84	400.6	
									≥85	707.5	Census-derived denominator that may not have fully matched catchment area.
	2017 – 2018							158.4	16 –49	29.9	
									50 –64	146.9	

For peer review only

									65 –74	310.4	Required specific symptoms and evidence of treatment and some CAP events may not have had this information documented
									75 –84	559.5	
									≥85	1522.6	
	2013 – 2018							120.4	--	--	
Thorrington 2019, BMC Med	2004 – 2005	England	Inpatients only	≥65 y	ICD 10 codes (1st position only): J18 (pneumonia of unspecified causative organism)	HAP Included	Mid-year population estimates for England for 2004 to 2015 from Office for National Statistics	NA	≥65	829	Incidence is per 100,000 person-years. Fewer ICD-10 codes included than other analyses
	2014 – 2015								≥65	1787	
Trotter 2008, EID	1997 – 1998	England	Inpatients only	≥65 y	ICD 10 codes (1st position only): J12 – J18	HAP Included	Mid-year population estimates for England for 1997 to 2004 from the Office for National Statistics	NA	65 –74	263	Incidence estimates converted to 100,000 population
									75 –84	684	
									≥85	1599	
	2004 – 2005								65 –74	355	
									75 –84	877	
									≥85	2218	
**Lower Respiratory Tract Infection**						**Pneumonia**					
Current Study	2018 – 2019	Bristol (Southmead Hospital)	Inpatients only	≥18 y	Clinical signs/symptoms of heart failure or elevated pro-NT BNP or radiological change **AND** Retrospective ICD-10 code analysis: J09, J10, J11; J20, J21, J22, J40, J41, J42, J44, J45, and J46	Excludes all Pneumonia	Based on number of persons ≥18 years registered in referring GP practices. For practices with split referral patterns, number adjusted for percent of admissions that came to Southmead.	802	18 –49	254	
									50 –64	448	
									65 –74	1,252	
								739	75 –84	2,442	
									≥85	6,116	
Lovering 2001, Clinical Micro. & Infection	1994 – 1996	Bristol (Southmead Hospital)	Inpatients only	≥16 y	LRTI episodes from four groups by ICD 9/10 codes: (1) CAP; (2) chest infection or acute exacerbation in presence of asthma; (3) chest infection or acute exacerbation in presence of COPD; or (4) bronchitis with no radiological evidence of pneumonia or pre-existing respiratory disease, such as COPD or asthma. No specified codes provided.	Includes Community-acquired Pneumonia	No information on denominator provided	623	16 –39	151	Incidence converted to per 100,000 population
									40 –49	175	
									50 –59	294	Study involved single hospital and no mention of source of denominator mentioned.
									60 –69	1,086	
									70 –79	2,135	
						HAP Excluded			>79	3,141	
Millet	1997 –	UK	Both	≥65 y	Read and ICD 10 codes; no specified	Includes	Mid-year UK population	12,293	65 –69	9,221	Incidence

For peer review only - http://bmjopen.bmj.com/site/about/guidelines.xhtml

2013, J Clin Epidemiol	2011		inpatients and outpatients		codes provided. For ICD-10, used first diagnosis code for inpatient episode only.	Community-acquired Pneumonia<br>HAP Excluded	estimates from Office for National Statistics. (Patients were not considered at risk for community-acquired LRTI during an LRTI illness-episode, during a HES hospitalization, or for 14 days after any HES hospitalization or CPRD hospital code. This person-time was excluded from denominator.)		70 –74<br>75 –79<br>80 –84<br>85 –89<br>≥90	10,740<br>12,607<br>15,137<br>18,791<br>26,287	converted to per 100,000 person years
**Heart Failure**						**All or First Episode**					
Current Study	2018-2019	Bristol (Southmead Hospital)	Inpatients only	≥18 y	Clinical signs/symptoms of heart failure or elevated pro-NT BNP or radiological change<br>**AND**<br>Retrospective ICD-10 code analysis: I110; I130; I132; I50	All	Based on number of persons ≥18 years registered in referring GP practices. For practices with split referral patterns, number adjusted for percent of admissions that came to Southmead.	328<br>402	18 –49<br>50 –64<br>65 –74<br>75 –84<br>≥85	21<br>206<br>869<br>1,645<br>4,005	
Uijl 2019, Eur J Heart Fail	2000 – 2010	UK	Both inpatients and outpatients	≥55 y	4 sources of HER were linked: CPRD primary care records, HES secondary care hospital charges, Myocardial Ischaemia National Audit Project (MINAP) disease registry, and ONS national death registry. HES ICD-10 codes: Heart failure: I110, I130, I132, I260, I50 and I21. Individuals were excluded if they presented a history of HF before their index date in CPRD, HES or MINAP.	First episode at 55 years or older counted	Not Reported	Not Reported	55 –64, M<br>55 –64, F<br>65 –74, M<br>65 –74, F<br>>75, M<br>>75, F	360<br>190<br>1,360<br>920<br>3,440<br>2,800	Incidence converted to per 100,000 person years<br>Included first episode of HF (inpatient or outpatient) at age 55 and up, so repeat episodes not included.

[a] Only the most recent incidence estimates from the Nottingham CAP study were included.
[b] Add text names for all listed ICD codes in this footnote or appendix.
[c] Included hospitals were Hull Royal Infirmary, Castle Hill Hospital, Princess Royal Hospital, Scarborough District General Hospital, Bridlington & District Hospital, York Teaching Hospital, Scunthorpe General Hospital, Goole & District Hospital.
[d] For the current study, only age-group estimates from the retrospective analysis were included in this table, but these were closely comparable to the annualized incidence estimates based on the prospective review. See Table 3 for full results.

For peer review only

Figure Legends:

Figure 1: aLRTD admissions identified by Retrospective ICD-10 diagnostic code analysis at North Bristol National Health Service Trust— United Kingdom 2018–2019.

Monthly number of patients admitted, based on ICD-10 coding analysis, with (A) all acute Lower Respiratory Tract Disease (aLRTD) (black bars), (B) Pneumonia (slashed bars), (C) Non-pneumonic Lower Respiratory Tract Infection (NP-LRTI) (white bars), (D) Other LRTD (cross-hash bars), and (E) Heart Failure (grey bars).

Figure 2: Flow diagram of the Prospective Review

For peer review only

For peer review only - http://bmjopen.bmj.com/site/about/guidelines.xhtml

For peer review only

For peer review only - http://bmjopen.bmj.com/site/about/guidelines.xhtml

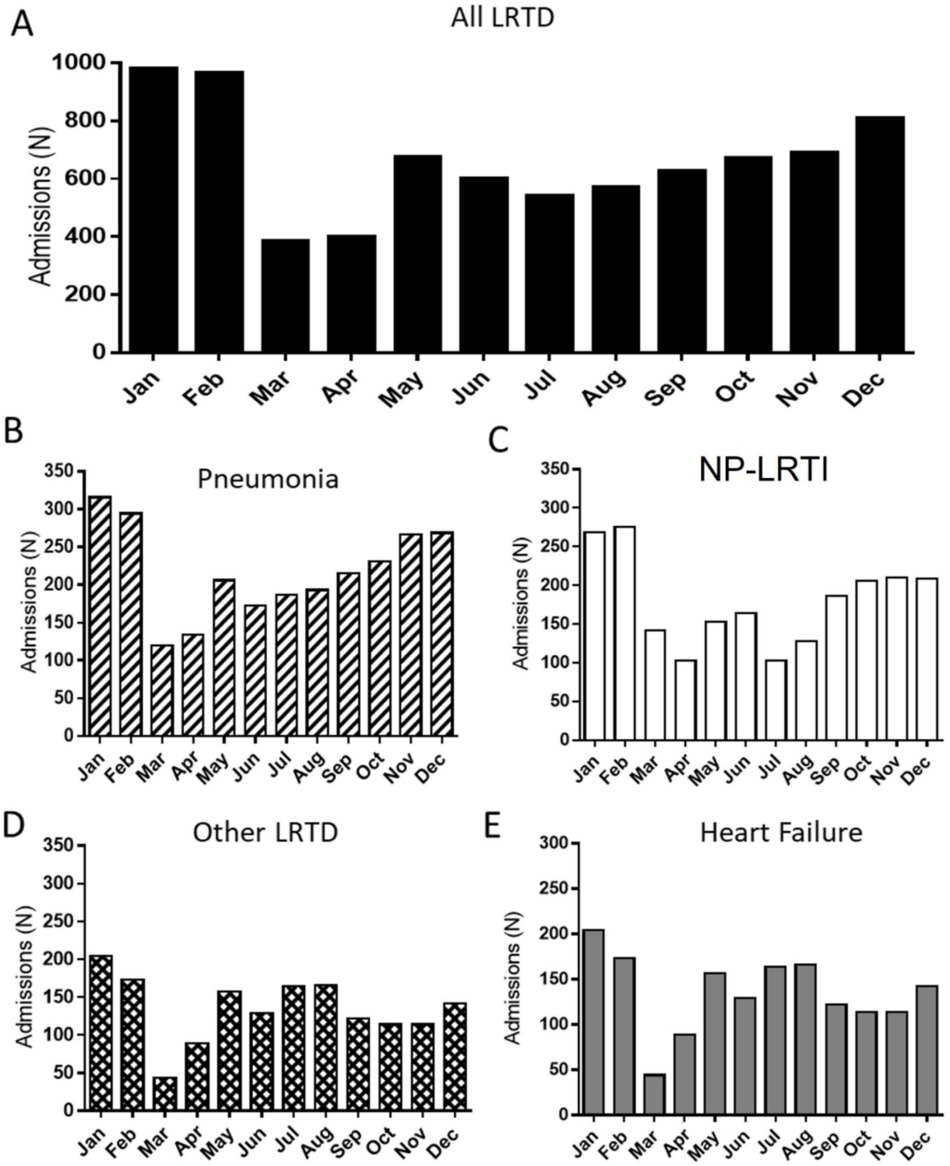

Figure 1: aLRTD admissions identified by Retrospective ICD-10 diagnostic code analysis at North Bristol National Health Service Trust— United Kingdom 2018–2019. Monthly number of patients admitted, based on ICD-10 coding analysis, with (A) all acute Lower Respiratory Tract Disease (aLRTD) (black bars), (B) Pneumonia (slashed bars), (C) Non-pneumonic Lower Respiratory Tract Infection (NP-LRTI) (white bars), (D) Other LRTD (cross-hash bars), and (E) Heart Failure (grey bars).

169x207mm (300 x 300 DPI)

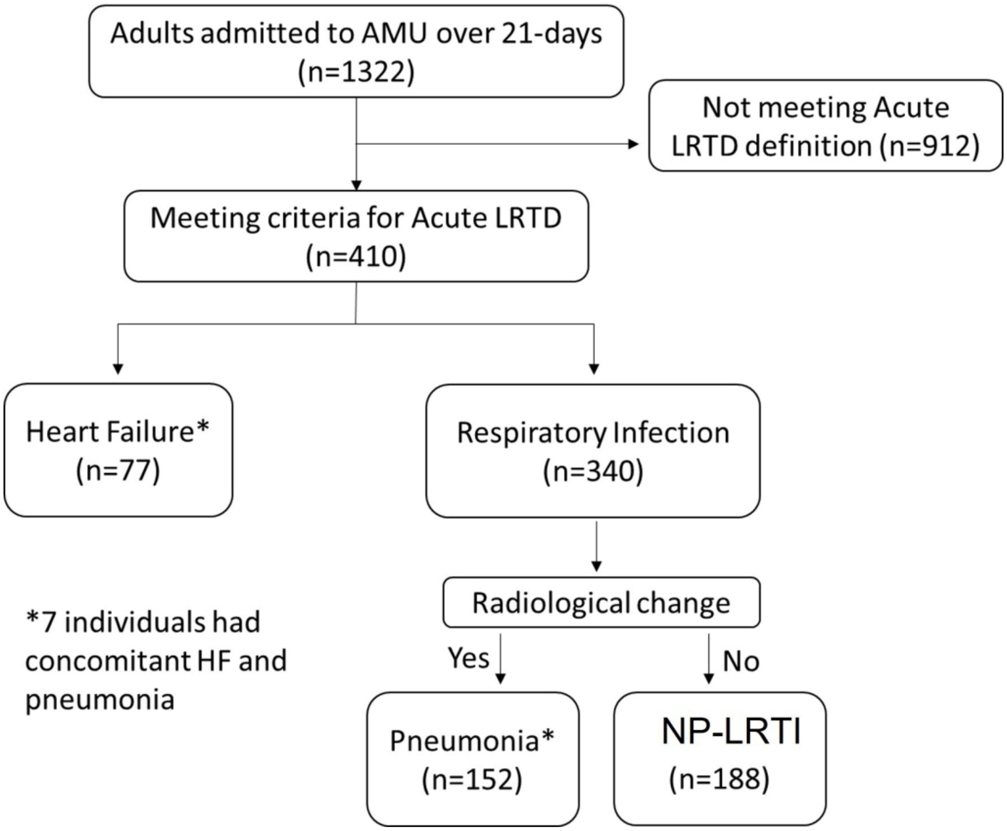

Figure 2: Flow diagram of the Prospective Review

149x123mm (300 x 300 DPI)

Supplementary Data 1: ICD-10 codes used for patient identification for Retrospective Analysis

ICD-10 code	Definition	Pneumonia	NP-LRTI	HF	Other aLRTD
J09	Influenza due to identified zoonotic or pandemic influenza virus		X		
J10	Influenza due to identified seasonal influenza virus		X		
J11	Influenza, virus not identified		X		
J12	Viral pneumonia, not elsewhere classified	X			
J13	Pneumonia due to *Streptococcus pneumoniae*	X			
J14	Pneumonia due to *Haemophilus influenzae*	X			
J15	Bacterial pneumonia, not elsewhere classified	X			
J16	Pneumonia due to other infectious organisms, not elsewhere classified	X			
J17	Pneumonia in diseases classified elsewhere	X			
J18	Pneumonia, organism unspecified	X			
J20	Acute bronchitis		X		
J21	Acute bronchiolitis		X		
J22	Unspecified acute lower respiratory infection		X		
J40	Bronchitis, not specified as acute or chronic		X		
J41	Simple and mucopurulent chronic bronchitis		X		
J42	Unspecified chronic bronchitis		X		
J43	Emphysema				X
J44	Other chronic obstructive pulmonary disease (including J44.0 chronic		J44.0 only		X (J44.0)

For peer review only

	obstructive pulmonary disease with acute lower respiratory infection)				
J45	Asthma				X
J46	Status asthmaticus				X
J47	Bronchiectasis				X
J85	Abscess of lung and mediastinum		X		
J86	Pyothorax		X		
J90	Pleural effusion, not elsewhere classified				X
J91	Pleural effusion in conditions classified elsewhere				X
J95	Postprocedural respiratory disorders, not elsewhere classified				X
J96	Respiratory failure, not elsewhere classified				X
J98	Other respiratory disorders				X
J99	Respiratory disorders in diseases classified elsewhere				X
I110	Hypertensive heart disease with heart failure			X	
I130	Hypertensive heart and chronic kidney disease with heart failure and stage 1 through stage 4 chronic kidney disease			X	
I132	Hypertensive heart and chronic kidney disease with heart failure and with stage 5 chronic kidney disease, or end stage renal disease			X	
I50	Heart failure			X	

For peer review only

Supplementary Data 2: Case Definitions

Condition	Definition	Reference
Acute Lower Respiratory Tract Disease (aLRTD)	Acute lower respiratory tract disease (aLRTD) encompasses pneumonia, non-pneumonic lower respiratory tract infection (LRTI), acute bronchitis, exacerbation of underlying respiratory disease including asthma and chronic obstructive pulmonary disease (COPD), and cardiac failure with respiratory symptoms.<br><br>Pneumothorax, pulmonary embolism, progression or new diagnosis of primary or secondary lung malignancy were excluded from aLRTD.	
Pneumonia	Pneumonia was defined as infection affecting the airways (below the level of the larynx), with either:<br>(1) an acute illness with radiographic shadowing which was at least segmental or present in more than one lobe and was not known to be previously present or due to other causes<br>(2) in the absence of radiological investigation, clinical confirmation of pneumonic disease in the opinion of the treating physician	Lim WS, Baudouin SV, George RC, *et al*; Pneumonia Guidelines Committee of the BTS Standards of Care Committee. BTS guidelines for the management of community acquired pneumonia in adults: update 2009. Thorax. 2009 Oct;64 Suppl 3:iii1-55.
Non-Radiologically Proven Lower Respiratory Tract Infection (NP-LRTI)	An infection that affects the airways (below the level of the larynx) including the trachea and alveoli, with neither the presence of radiological change nor a clinical diagnosis of pneumonia from the treating physician, i.e. non-pneumonic infection in the lungs.	Anderson W, Winter J. Managing LRTI in adults in the community. Practitioner. 2009 Nov;253(1723):21-5, 2-3. PMID: 20043506.
Cardiac/Heart Failure (HF)	A clinical syndrome with symptoms and/or signs caused by a structural and/or functional cardiac abnormality and corroborated by elevated natriuretic peptide levels and or objective evidence of pulmonary or systemic congestion.	Bozkurt, Biykem et al.Universal Definition and Classification of Heart Failure. Journal of Cardiac Failure, Volume 27, Issue 4, 387 – 413.
Other aLRTD	aLRTD which was neither pneumonia, NP-LRTI nor HF was classified as 'Other aLRTD'. This therefore includes non-infective exacerbations of chronic respiratory disease such as asthma, COPD and bronchiectasis	

For peer review only

STROBE Statement—checklist of items that should be included in reports of observational studies

	Item No	Recommendation	Page No
**Title and abstract**	1	(*a*) Indicate the study's design with a commonly used term in the title or the abstract	3
		(*b*) Provide in the abstract an informative and balanced summary of what was done and what was found	3
**Introduction**			
Background/rationale	2	Explain the scientific background and rationale for the investigation being reported	5-6
Objectives	3	State specific objectives, including any prespecified hypotheses	6
**Methods**			
Study design	4	Present key elements of study design early in the paper	7
Setting	5	Describe the setting, locations, and relevant dates, including periods of recruitment, exposure, follow-up, and data collection	7-9
Participants	6	(*a*) *Cohort study*—Give the eligibility criteria, and the sources and methods of selection of participants. Describe methods of follow-up *Case-control study*—Give the eligibility criteria, and the sources and methods of case ascertainment and control selection. Give the rationale for the choice of cases and controls *Cross-sectional study*—Give the eligibility criteria, and the sources and methods of selection of participants	7-9
		(*b*) *Cohort study*—For matched studies, give matching criteria and number of exposed and unexposed *Case-control study*—For matched studies, give matching criteria and the number of controls per case	
Variables	7	Clearly define all outcomes, exposures, predictors, potential confounders, and effect modifiers. Give diagnostic criteria, if applicable	8
Data sources/ measurement	8*	For each variable of interest, give sources of data and details of methods of assessment (measurement). Describe comparability of assessment methods if there is more than one group	7-8
Bias	9	Describe any efforts to address potential sources of bias	15-16
Study size	10	Explain how the study size was arrived at	7-8
Quantitative variables	11	Explain how quantitative variables were handled in the analyses. If applicable, describe which groupings were chosen and why	8-9
Statistical methods	12	(*a*) Describe all statistical methods, including those used to control for confounding	9
		(*b*) Describe any methods used to examine subgroups and interactions	NA
		(*c*) Explain how missing data were addressed	NA
		(*d*) *Cohort study*—If applicable, explain how loss to follow-up was addressed *Case-control study*—If applicable, explain how matching of cases and controls was addressed *Cross-sectional study*—If applicable, describe analytical methods taking account of sampling strategy	NA
		(*e*) Describe any sensitivity analyses	NA

Continued on next page

For peer review only - http://bmjopen.bmj.com/site/about/guidelines.xhtml

**Results**			
Participants	13*	(a) Report numbers of individuals at each stage of study—eg numbers potentially eligible, examined for eligibility, confirmed eligible, included in the study, completing follow-up, and analysed	10, 21-24
		(b) Give reasons for non-participation at each stage	NA
		(c) Consider use of a flow diagram	24
Descriptive data	14*	(a) Give characteristics of study participants (eg demographic, clinical, social) and information on exposures and potential confounders	21-24
		(b) Indicate number of participants with missing data for each variable of interest	NA
		(c) *Cohort study*—Summarise follow-up time (eg, average and total amount)	NA
Outcome data	15*	*Cohort study*—Report numbers of outcome events or summary measures over time	10-11, 21-24
		Case-control study—Report numbers in each exposure category, or summary measures of exposure	*NA*
		Cross-sectional study—Report numbers of outcome events or summary measures	*NA*
Main results	16	(*a*) Give unadjusted estimates and, if applicable, confounder-adjusted estimates and their precision (eg, 95% confidence interval). Make clear which confounders were adjusted for and why they were included	11
		(*b*) Report category boundaries when continuous variables were categorized	NA
		(*c*) If relevant, consider translating estimates of relative risk into absolute risk for a meaningful time period	NA
Other analyses	17	Report other analyses done—eg analyses of subgroups and interactions, and sensitivity analyses	10, 21-25
**Discussion**			
Key results	18	Summarise key results with reference to study objectives	12
Limitations	19	Discuss limitations of the study, taking into account sources of potential bias or imprecision. Discuss both direction and magnitude of any potential bias	15
Interpretation	20	Give a cautious overall interpretation of results considering objectives, limitations, multiplicity of analyses, results from similar studies, and other relevant evidence	12-16
Generalisability	21	Discuss the generalisability (external validity) of the study results	12-16
**Other information**			
Funding	22	Give the source of funding and the role of the funders for the present study and, if applicable, for the original study on which the present article is based	1

*Give information separately for cases and controls in case-control studies and, if applicable, for exposed and unexposed groups in cohort and cross-sectional studies.

Note: An Explanation and Elaboration article discusses each checklist item and gives methodological background and published examples of transparent reporting. The STROBE checklist is best used in conjunction with this article (freely available on the Web sites of PLoS Medicine at http://www.plosmedicine.org/, Annals of Internal Medicine at http://www.annals.org/, and Epidemiology at http://www.epidem.com/). Information on the STROBE Initiative is available at www.strobe-statement.org.

For peer review only
